# Exploring the therapeutic potential of oleanolic acid and its derivatives in cancer treatment: a comprehensive review

**DOI:** 10.1007/s13205-025-04209-5

**Published:** 2025-02-07

**Authors:** Rachel Savio D’Mello, Vividh Mendon, Padmini Pai, Ipshita Das, Babitha Kampa Sundara

**Affiliations:** 1 Manipal School of Life Sciences, Manipal Academy of Higher Education, Manipal, Karnataka 576104 India; 2 Department of Biophysics, Manipal School of Life Sciences, Manipal Academy of Higher Education, Manipal, Karnataka 576104 India

**Keywords:** Anticancer, Apoptosis, Inhibitors, Oleanolic acid, Pathways

## Abstract

Oleanolic acid (OA) is a triterpenoid that occurs naturally and may be isolated from various plants. Analogs of oleanolic acid can be produced artificially or naturally. The current treatments have limited selectivity and may also impact normal cells. OA and its derivatives provide a promising cancer treatment platform with greater selectivity and less toxic effects. As a result of their enhanced sensitivity, selectivity, and low toxicity, they are great options for focusing on particular biological pathways and reducing the growth of tumor cells. The effects of OA and derivatives of OA on various cancer types have been investigated. However, breast and hepatocellular malignancies are the most studied cancers. In breast cancer, derivatives such as saikosaponin A (SSa), saikosaponin B (SSb), and SZC014 influence key pathways such as the Janus kinase/signal transducer and activator of transcription (JAK/STAT), protein kinase-B (Akt), and nuclear factor-kappa B (NF-κB) pathways, inhibiting metastasis, angiogenesis, and cell migration, respectively. When a para-aminobenzoic acid (PABA)/nitric oxide (NO) derivative of OA is administered to HepG2 cells, the reactive oxygen species (ROS)/mitogen-activated protein kinase (MAPK)-mediated mitochondrial pathway causes apoptosis. Nanoformulations incorporating OA, such as OA-paclitaxel (PTX), show potential for suppressing tumor progression by inhibiting drug efflux mechanisms. Thus, exploring the interactions of OA and a few of its derivatives with various cellular pathways offers a promising approach to combating different types of cancer. This review delves into the potential of oleanolic acid and its derivatives in retarding cancer progression through their interactions with diverse cellular pathways.

## Introduction

**Fig. 1:**
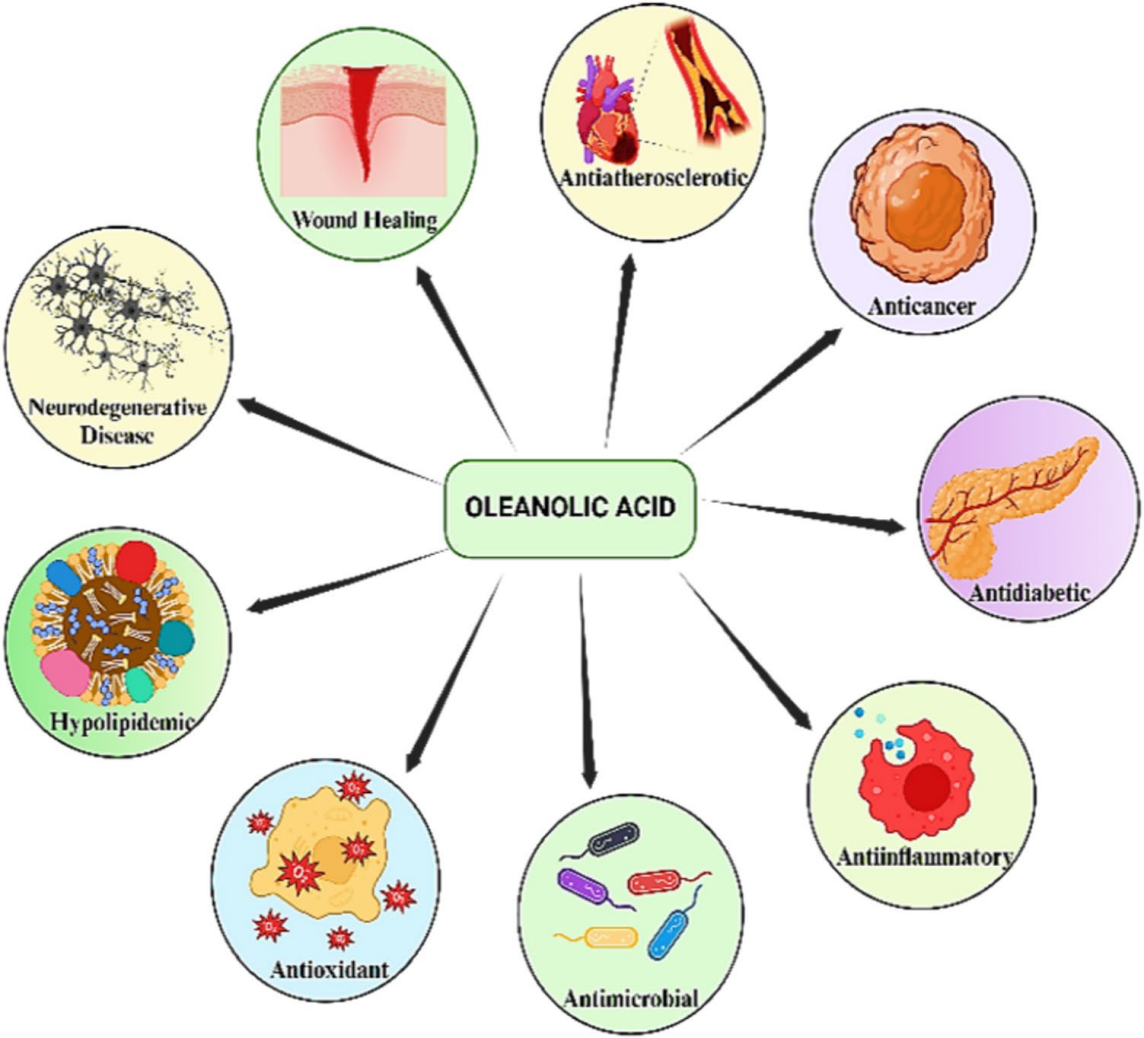
Role of oleanolic acid in the treatment of diseases such as Alzheimer's, hyperglycemia, diabetes, atopic dermatitis, wound healing, and antimicrobial activity

Triterpenoid oleanolic acid (3β-hydroxy-olea-12-en-28-oic acid) (OA) is extracted from more than 1600 different plants. It demonstrates anti-inflammatory, antioxidant, anticancer, antimicrobial, and many other properties, as shown in Fig. 1 (Han et al. 2021). It can be found in nature as an aglycone of triterpenoid saponins or as a free acid. It is commonly present with its isoforms such as ursolic acid (UA), moronic acid, or betulinic acid (Kang et al. 2020). OA is sourced from clove leaves, pomace, clove blossoms, mistletoe sprouts, and several other plants (Kang et al. 2017). By activating the Akt/Foxg1 pathway, saikosaponin D (SSd), a naturally occurring triterpenoid derived from *Bupleurum falcatum L.*, induces the downregulation of neurogenesis and cognitive impairment (Lixing et al. 2017). *Radix Bupleuri* is separated to produce Saikosaponin A (SSa). According to Liu et al. (2018b), it lessens heart fibrosis and decreases cardiac dysfunction. Many different plants contain the triterpene pomolic acid, which helps to fight glioblastoma and tumors via apoptosis. (Guimarães et al. 2017). According to Zhang et al. (2017), 2-cyano-3,12-dioxooleana-1,9(11)-dien-28-oic acid ethyl amide (CDDO-Ea) has a cytoprotective impact by attenuating the accumulation of ROS and increasing heme oxygenase-1 (HO-1) activity. Bardoxolone methyl substituted with an α-cyano-substituted α, βunsaturated ketone (CUK) in its ring has greater therapeutic efficacy and safety than its parent molecule (Huang et al. 2017).

OA is vital in the treatment of Alzheimer’s, hyperglycemia, diabetes, atopic dermatitis, wound healing, and antimicrobial activity (Fig. 1). According to Han et al. (2021), OA protects microglia from Alzheimer’s disease and reduces cognitive decline. The inflammatory response induced by allergies is reduced by OA through the release of cytokines caused by mast cells (Kang et al. 2020). According to Kang et al. (2017), OA exploits the peroxisome proliferator-activated receptor ʽ (PPAR̔di as an antagonist to prevent cartilage deterioration caused by hyperglycemia. It provides protection against renal oxidative stress and kidney damage and can be utilized to treat diet-induced prediabetes (Gamede et al. 2021). By lowering the levels of high-mobility group box 1 (HMGB1), OA exhibits a hepatoprotective effect (Wang et al. 2019c). To ameliorate atopic dermatitis, it inhibits the Akt, Nuclear Factor kappa light-chain-enhancer of activated B cells (NF-κB), and Signal Transducer and Activator of Transcription 1 (STAT1) signaling pathways (Kang et al. 2021).

Globally, nearly 10 million deaths due to cancer were recorded in 2020 (Debela et al. 2021). Cancer treatments often include surgery, radiation, and chemotherapy. However, these methods are limited by the potential negative impact on native cells and the potential for resistance to arise with continued use. Techniques to reduce harm and increase therapeutic sensitivity are being developed to address these issues (Debela et al. 2021).

Anticancer drug discovery was revolutionized with the use of natural products. Irinotecan, vincristine, paclitaxel, and etoposide are a few plant-derived anticancer drugs whereas actinomycin D and mitomycin C are bacteria-derived. As the landscape of cancer therapy is expanding, new opportunities are being sought for the development of natural anticancer drugs and for exploring their utility as therapeutics (Huang et al. 2021). The compound OA and its derivatives are relatively nontoxic. They are crucial components for cancer therapy since they can interact with a variety of biological processes. In triple-negative breast cancer (TNBC), SSd inhibits proliferation by inducing apoptosis via the Wingless-related integration site (Wnt)/β-catenin pathway (Wang et al. 2018). When combined with aspirin, OA oxime morpholide derivatives decrease cytotoxicity, downregulate NF-kB levels, and aid in the prevention of liver cancer (Krajka-Kuźniak et al. 2019b). When treating non-small cell lung cancer, UA and OA may target the p62 protein and mitophagy (Castrejón-Jiménez et al. 2019). OA triggers apoptosis in melanoma cells via the NF-kB pathway (Woo et al. 2021). It inhibits tumor initiation and development by affecting phosphatidylinositide 3-kinase (P13K)/Akt/mammalian target of rapamycin (mTOR)/NF-kB signaling pathways. It activates autophagy via the extracellular signal-regulated kinase/c-Jun N-terminal kinase/mitogen-activated protein kinase (ERK/JNK/p38 MAPK) signaling pathways. SZC015, a derivative of OA, activates an intrinsic apoptotic pathway by up-regulating poly (ADP-ribose) polymerase (PARP), caspase 3 and 9, and increases Bcl-2 Associated X-protein (Bax)/Bcl2 expression. OA suppresses angiogenesis by inhibiting the expression of STAT3 and sonic hedgehog signaling pathways (Žiberna et al. 2017). Multiple such examples demonstrate that OA and its derivatives can act as potent anti-cancer moieties.

This review focuses on several cellular pathways that OA and its derivatives use to induce apoptosis and slow cancer growth. These advancements might help create more effective and innovative. These advancements might help create more effective and innovative therapeutic approaches.

## Breast cancer

Breast cancer is more prevalent among women and ranks 5th in cancer-related deaths. As of 2020, GLOBOCAN reported about 2.3 million fatalities. The World Health Organization states that approximately 18 different types of histological breast cancer are known. Some of the frequently used therapeutic strategies include surgery, radiation therapy, and endocrine therapy (Łukasiewicz et al. 2021).

Achyranthoside H methyl ester (AH-Me), a derivative of OA shows increased cytotoxicity towards MCF-7 and MDA-MB-453 cells. 1-[2-cyano-3-,12-dioxooleana-1,9(11)-dien-28-oyl] imidazole (CDDO-Im) causes DNA damage via ROS production and thus induces apoptosis in BRCA1 null and estrogen receptor-negative breast cancer cells (Fukumura et al. 2009). CDDO-Im downregulates the growth and metastasis of breast cancer by blocking the Epidermal growth factor receptor (EGFR)/Signal Transducer and Activator of Transcription 3 (STAT3)/Sox-2 pathways in Tumor-associated macrophages (TAMs) (Yang et al. 2013).

Chu and colleagues studied the effect of SZC014, on Breast cancer cells. SZC014 has anticancer effects by impairing the cell cycle, inhibiting inflammation, and inhibiting cell death, which are linked to the Akt and NF-kB pathways. SZC014 decreases breast cancer cell viability and displays minimal toxicity towards mammalian epithelial cancer cell lines, showing that it has greater specificity for breast cancer cells. SZC014 reduced the viability of these cells more strongly than OA. It suppresses cell migration and colony formation. Cell fragmentation and shrinkage of apoptotic cells were observed after SZC014 treatment. When the Bax/Bcl2 ratio increases, procaspase 3 and procaspase 9 levels are suppressed, and Poly (Adenosine Diphosphate-ribose) polymerases (PARP) cleavage increases after the cells are treated with SZC014. This causes G1 phase arrest in the cells. The Akt pathway is a dominant target of SZC014. SZC014 inhibits the activity of cyclooxygenase 2 (COX-2), a protein that promotes angiogenesis and cancer cell proliferation. SZC014 inhibits the nuclear translocation of p65. N-acetylcysteine (NAC), an active scavenger of SZC014 inhibits SZC014-induced apoptosis (Chu et al. 2017). Cheng et al. described another OA derivative namely D Rhamnose-Hedrin (3b-[a-l-arabinopyranosyl)-oxy] olean-12-en-28-oicacid) (DRβ-H). This is an oleanane type of triterpenoid with anticancer properties. The RNA binding region (RNP1, RRM) containing (RNPC1), an RNA-binding protein, is attenuated in highly invasive breast cancer. DRβ-H showed cytotoxic effects at higher concentrations. It increased the levels of RNPC1 and E-cadherin after 24 h of treatment shown by western blotting. Knockdown of RNPC1 reduced the upregulation of these two proteins, after DRβ-H treatment. It also diminishes the antimetastatic activities of DRβ-H (Cheng et al. 2017).

P-glycoproteins (P-gp), ATP-driven transmembrane transporters, are found at relatively high concentrations in multiple types of cancers. The side effects of P-gp inhibitors limit their application in cancer treatment. *Bupleurum chinense DC* (BCDC) inhibits P-gp and Multidrug resistance 1 (MDR1)/mRNA. SSd is used to reverse multidrug resistance (MDR). MCF-7/Adriamycin (ADR) cells are affected concurrently with SSd and ADR. SSd downregulates the expression of P-gp and MDRI mRNA. SSd restores the accumulation of Rhodamine 123 (Rh123), which demonstrates that the reversal of MDR by SSd is due to its effect on P-gp-mediated transport (Li et al. 2017). In TNBC, the progesterone receptor, human epidermal growth factor receptor 2 (HER2), and estrogen receptor are negatively expressed. SSd inhibits the proliferation of HCC1937 cells and represses TNBC cell line proliferation has inhibitory effects, however, SSb and SSc show less cytotoxicity, indicating that the configuration of the C-16 position is important for the cytotoxic effect of sapogenin. Treatment with SSd increased nuclear condensation, programmed-cell death, and cleaved caspase 3 levels. Cotreatment with SSd and carbobenzoxy-valyl-alanyl-aspartyl-[O-methyl]-fluoromethylketone (z-VAD-fmk) an inhibitor of caspase, decreased cleaved caspase 3 and PARP levels to a greater extent than treatment with SSd alone, suggesting that SSd causes apoptosis. The inhibitory effect of SSd is due to its impact on the Wnt/β-catenin pathway. Nuclear and cytoplasmic fractions show reduced β-catenin levels. Cotreatment with SSd and Lithium chloride, an activator of β-catenin increases cell viability, demonstrating that the effect of SSd is mediated by a reduction in β-catenin (Wang et al. 2018).

Another study showed that cell adhesion molecules (CAMs), which belong to the integrin family, promote invasion and adhesion of cells. In addition, CAMs are overexpressed during the invasion and proliferation of cells. The FERM fragment integrin subunit of focal adhesion kinase (FAK) assists in these processes. FAK assists in the advancement of breast cancer. Methyl 3-hydroxyimino-11-oxoolean-12-en28-oate (HIMOXOL) and 12α-bromo-3-hydroxyimonoolean28 13-olide (Br-HIMOLID) attenuate cell metabolism and show greater cytotoxicity than the parent compound. These compounds decrease cancer cell migration and invasion potential. HIMOXOL and Br-HIMOLID also decreased the expression of integrin beta-1 (ITGB1) and integrin 1. Br-HIMOLID downregulates FAK and its phosphorylated forms. The antimigratory effect of OA analogs is due to their ability to downregulate proteins such as ITGB1, protein tyrosine kinase 2 (PTK2), and the Paxillin (PXN) gene (Lisiak et al. 2017). Another study showed that HER2 is overexpressed in breast cancers. HIMOXOL and Br-HIMOLID, semisynthetic analogues of OA, induce cancer cell death through apoptosis and autophagy. These analogs are modified at the C3, C11, and C28 positions. HIMOXOL and Br-HIMOLID reduced cell viability to a greater extent than the parent moiety. Br-HIMOLID was toxic only after 3 days of incubation. HIMOXOL and Br-HIMOLID decreased colony-forming ability at all concentrations. Cell cycle analysis of SK-BR-3 cells revealed that OA treatment caused stagnation at the G0/G1 phase and decreased the number of cells in the S phase. Br-HIMOLID increases the number of apoptotic cells. Bax, PARP, and cyclin D levels increase at higher concentrations of Br-HIMOLID. Br-HIMOLID increases LC3-II/LC3-I ratio, the mammalian target of rapamycin (mTOR) negatively regulates autophagy, which is marked by decreased levels of p62. OA, HIMOXOL, and Br-HIMOLID attenuate cell migration (Lisiak et al. 2021).

*Lantana camara* is a medicinal plant from which triterpenoids such as Lantadene A, B, and C are derived. These compounds demonstrate antimicrobial, anti-insecticidal, and antifungal properties. Lantadene A and B show elevated radical scavenging activity. Both compounds decreased the viability of MCF-7 cells. Lantadene C and icterogenin also demonstrate anti-proliferative activity. Lantadene B is important for cell cycle arrest and caspase 9 induction. Lantadene B decreases cells in the G2/M phase while increasing cells in the early stages of the cell cycle (Shamsee et al. 2018).

Esculentoside A (EsA) is a triterpene that shows anti-inflammatory activity by targeting S3a, a ribosomal protein. It also shows anti-proliferative and anti-metastatic potential. It impairs tumor growth by inhibiting cancer stem cells. Experimental mammary tumor-6 (EMT-6) mammosphere cells exhibit stem cell properties and can be used as a CSC anticancer target. EsA at increasing concentrations inhibits mammosphere formation in murine and breast cancer cells. Apoptotic cells increased following EsA treatment. EsA significantly reduces the expression of proteins that confer stem-like properties. The levels of proteins that antagonize apoptosis decrease, and the levels of proteins that assist in apoptosis increase after EsA treatment. It also downregulates IL-6, pSTAT3, and STAT3 expression (Liu et al. 2018a).

STAT3 helps promote metastasis by mediating the Janus kinase/signal transducer and activator of the transcription (JAK/STAT) pathway. Activated STAT3 promotes matrix metallopeptidase 3 (MMP3) expression. Saikosaponin (SS), a triterpenoid, is responsible for the pharmacological activities of *Bupleurum.* STAT3 and vasodilator-stimulated phosphoprotein (VASP) show elevated expression in cancer cells. Treatment with SSb reduces the levels of c-myc and cyclin D1, which are involved in the STAT3 pathway and are regulators of cell proliferation. pSTAT3 and VASP expression also decreased in the MCF-7 cell line and cells with NSC74859. The VASP level increases when STAT3 is phosphorylated by IL6. Treatment of cells with ministered with SSb decreases MMP protein expression (Ma et al. 2019).

**Fig. 2:**
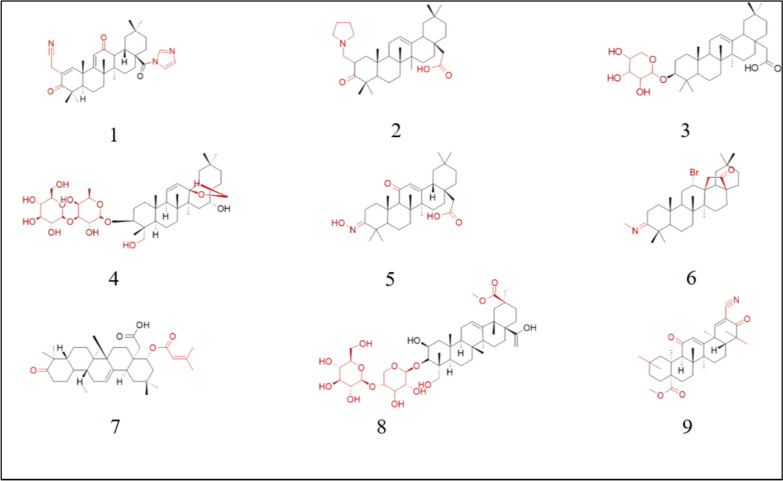
Structures of OA derivatives effective against breast cancer. 1: CDDO-Im; 2: SZC014; 3: DRβ-H; 4: SSd; 5: HIMOXOL; 6: BrHIMOXOL; 7: Lantadene B; 8: Esculentoside A; 9: CDDO-Me

Another study showed that TAMs assist in tumor progression and cell survival. TAMs recruit monocytes to tumor sites via chemokines and promote angiogenesis. TAMs suppress T cell expression and prevent their survival, augmenting immunosuppression in the tumor environment. Methyl-2-cyano-3,12-dioxooleana-1,9(11)-dien-28-oate (CDDO-Me) inhibits TAM infiltration into the tumor environment, prevents the secretion of chemokines, and elevates Tumor necrosis factor alpha (TNF-α) expression. It also increases the CD8 + :CD4 + T-cell ratio. CDDO-Me decreases the expression of TAM markers, such as CD206 and CD115. It reduces proangiogenic and IL-6 levels while increasing TNF-α expression. The activity of the chemokine (C-X-C motif) ligand 16 (CXCL16), increases, and the levels of chemokines such as chemokine ligand 2 (CCL2) decrease. The levels of matrix metalloproteinases (MMP2) and Arg1, which assist immunosuppressive TAMs, are attenuated after treatment with CDDO-Me. CDDO-Me also increases IL-1β levels, which assist in T-cell activation. CD3 + cells positive for the CD8 marker were significantly increased. After treatment with CDDO-Me, the spleens of mice exhibit reduced numbers of CD3 + CD4 + cells and increased numbers of CD3 + CD8 + cells (Ball et al. 2020). Zhou and his group studied CDDO-Me and investigated how it affects the Wnt/β-catenin pathway. The Wnt/β-catenin pathway is often dysregulated in multiple cancer types, including breast cancer. CDDO-Me targets nuclear factor erythroid-2 related factor-2, JAK/STAT, NF-kB, etc. Treatment of tumor cells with CDDO-Me attenuates tumor growth and inhibits the Wnt/β-catenin pathway. OA, CDDO, and CDDO-Me antagonize this pathway. CDDO-Me reduces the transcriptional activity of proteins that assist in signalling pathways. It may also act on the upstream region of Dishevelled Segment Polarity Protein 2 (DVL2) and β-catenin. When CDDO-Me is used, cells treated with Wnt exhibit decreased levels of phosphorylated low-density lipoprotein receptor-related protein 6 (pLRP6), pDVL2, LRP6, DVL2, β-catenin, etc. CDDO-Me reduces Wnt target gene expression. Its effect on LRP6 and Frizzled class receptor 7 (FZD7) does not depend on transcriptional regulation but relies on the lysosomal pathway. Glycogen synthase kinase 3 (GSK3) is not essential for the effect of CDDO-Me on signaling pathways. CDDO-Me reduces breast cancer cell viability and attenuates tumor sphere formation (Zhou et al. 2020a) (Fig. 2) (Table 1).

**Table 1:** OA derivative and its effect on breast cancer

Name of the derivative	IC50	Pathway/gene/RNA involved	Source	References
1-CDDO-Im	10–30 µM	EGFR/STAT3/Sox-2 pathways	Synthetic	(Hyer et al. 2005, Kim et al. 2011, So et al. 2013, Yand et al., 2013)
2-SZC014	9.8 µM	Akt pathway NF-kB pathway	Synthesized	(Chu et al. 2017)
3-DRβ-H	> 20 µM	RNPC1 proteins E-cadherin proteins	*Clematis ganpiniana*	(Cheng et al. 2017)
4-SSd	13.06 µM	P-gp Wnt/β-catenin pathway, PARP	*Bupleurum chinense*	(Li et al. 2017) (Wang et al. 2018)
5-HIMOXOL 6-BrHIMOLID	4.25 µM 5.81 µM	ITGB1 protein Integrin 1 protein	Semi-synthetic	(Lisiak et al. 2017) (Lisiak et al. 2021)
7-Lantadene B	112.2 µM	Bax, PARP, p62, and cyclin D levels	*Lantana camara*	(Shamsee et al. 2018)
8-Esculentoside A	177.68 µM	IL-6 protein PSTAT3 protein STAT3 protein	Root of* Phytolacca esculenta*	(Liu et al. 2018a)
9-CDDO-Me	0.51 µM	Wnt/β-catenin pathway, CXCL16, CCL2, IL-6	Synthetic	(Zhou et al. 2020a) (Ball et al. 2020)
OA-PTX nanoparticle	7.66 $\mu \text{M}$	P-gp	Synthetic	(Bao et al. 2020)

## Cervical cancer

In developing countries, cervical cancer is common among women. Most cases are found in developing countries. Cervical cancer mostly results from long-term infection with human papillomavirus (HPV). HPV16 and 18 cause 70% of cancer cases. New molecules and therapies are being discovered as treatments for cervical cancer (Edathara et al. 2020).

Epifriedelinol is a compound that shows excellent anti-inflammatory and antioxidant properties. It is isolated from *Aster tataricus’* roots and *Vitex penduncularis’s* leaves. Yang and colleagues observed the effects of epifriedelinol on C-33A and HeLa cell lines. The cell viability decreased with increasing concentrations of epifriedelinol. It also elevated caspase 3, 8, and 9 enzyme activity and cytosolic cytochrome c in both cell lines. Epifriedelinol also affects how the B-cell lymphoma 2 (Bcl-2) family of proteins is expressed, increasing the activity of Bad, Bim, and Bak proteins, and reducing the activity of others, such as the Bcl-xL and Bcl2 proteins. It also downregulates the expression of inhibitors of apoptosis proteins (Yang et al. 2017). Potočnjak and colleagues studied the combinatorial effect of cisplatin and OA. Cisplatin is a commonly employed agent for treating a wide range of cancers. However, it can cause cardiotoxicity, nephrotoxicity, and hepatotoxicity. Since the kidney is the major route of elimination, cisplatin often accumulates there and causes damage to the kidneys through ROS production. OA aids in repairing this damage and enhances the cervical cancer cells’ sensitivity to cisplatin. Cisplatin-treated mice exhibited elevated kidney damage and increased creatinine and blood urea nitrogen (BUN) concentrations in the serum, and these effects were attenuated by treatment with increasing concentrations of OA. OA attenuates 4-hydroxynonenal (4-HNE) and HO-1 levels, which are oxidative stress markers and prevents the loss of superoxide dismutase (SOD) activity. Higher doses of OA decreased tumor necrosis factor-alpha (TNF-α), autophagy-related gene 5 (Atg5), and light chain 3B (LC3B-I/II) levels in cisplatin-treated mice. OA also decreases the levels of p21 and results in limited caspase 3 and 9 cleavage and PARP in the kidney. Higher doses of OA decreased the expression of Extracellular signal-regulated kinases (ERK) and the IC50 values. Treating HeLa cells with OA and cisplatin suppresses the expression of the Bcl-2 protein while increasing LC3B-II expression (Potočnjak et al. 2019).

**Fig. 3:**
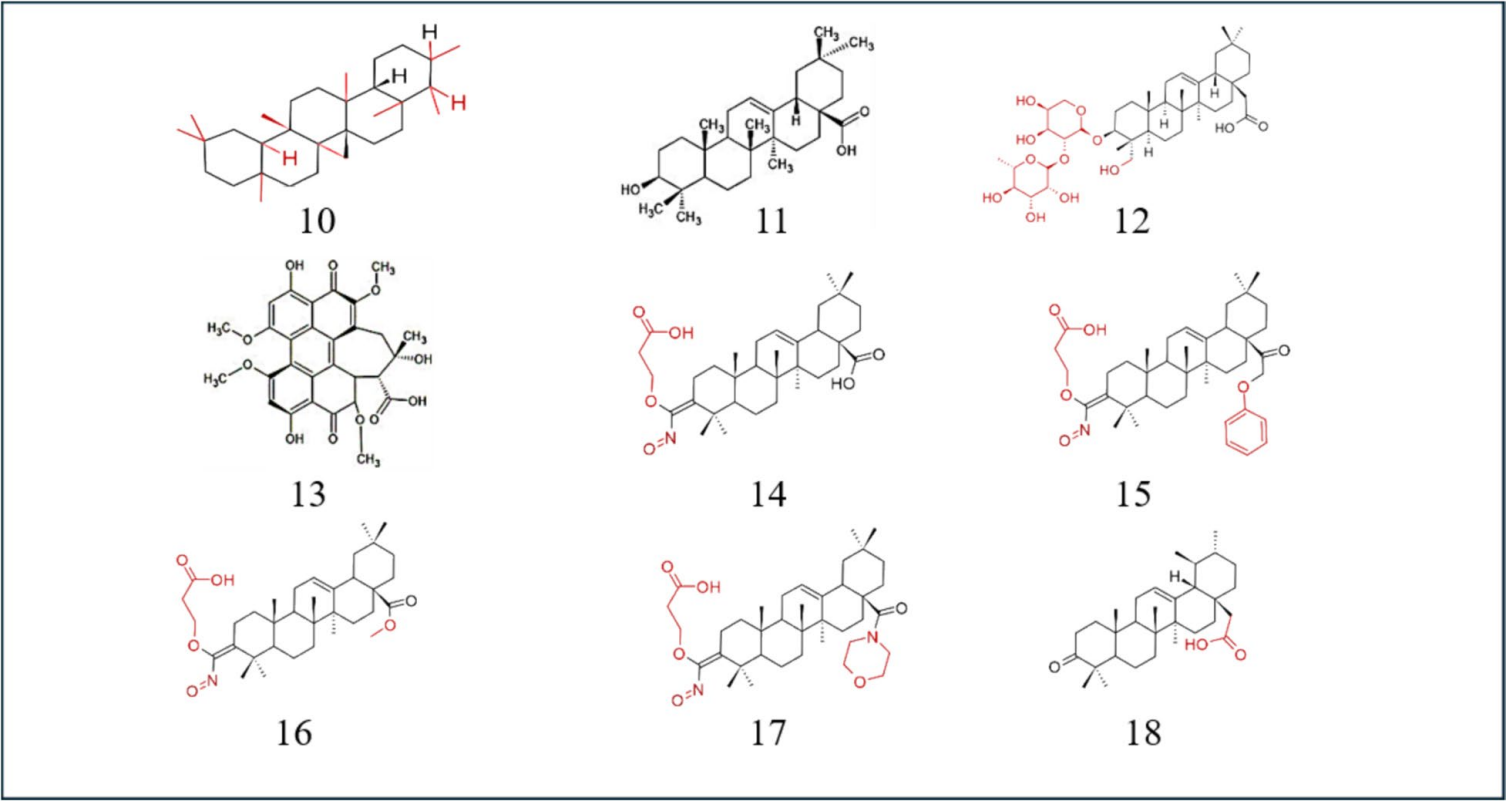
Structures of OA derivatives effective against cervical and hepatocellular cancer. 10: epifriedelinol; 11: OA; 12: α-hedrin; 13: HA; 14: SMAA; 15: SMAE; 16: SMAEM; 17: SMAM; 18: ursolic acid

Edathara and colleagues studied the effects of OA and esculetin on HeLa cell lines. The cytotoxic effects on the cell line were studied using a sulforhodamine B (SRB) assay at different concentrations of OA and esculetin. These genes affect the expression of cell cycle regulatory genes. Beta-galactosidase (β-gal) assay revealed that they were able to increase cell senescence, and semiquantitative polymerase chain reaction (PCR) results showed that OA and esculetin elevated tumor suppressor gene expression and downregulated proliferation regulator levels. Wound healing assays revealed that the migratory activity of cells decreased, and Wnt signalling-associated gene expression also significantly decreased in cells treated with OA and esculetin. Annexin V-Fluorescein isothiocyanate (FITC)/PI staining revealed that most of the cells treated with a combination of the two agents underwent apoptosis. Treatment with OA and esculetin led to the localization of the p16 and p53 genes in the cytoplasmic and perinuclear regions, revealing their apoptotic effects (Edathara et al. 2020). Ferroptosis is an essential process that prevents and kills tumor growth, especially in kidney and liver cancer. Therefore, the induction of ferroptosis in tumor cells is an essential therapeutic strategy. Ferroptosis is regulated by the Acyl-CoA synthase long-chain family member 4 (ACSL4) gene. OA treatment causes the accumulation of Fe2 + and malondialdehyde (MDA) and a reduction in tumor size. Glutathione (GSH) levels also decreased in mice treated with OA. With increasing concentrations of OA, HeLa cell viability decreases, and ROS levels increase. OA treatment increased ACSL4 and transferrin receptor 1 (TfR1) activity and decreased ferritin heavy chain (FTH1) and glutathione peroxidase 4 (GPX4) expression in both tumors and HeLa cells. Inhibiting the expression of ACSL4 reduced OA-mediated ferroptosis in this cell line (Xiaofei et al. 2021) (Table 2) (Fig. 3).

**Table 2:** OA derivative and its effect on cervical cancer

Name and structure of the derivative	IC50	Pathway/gene/RNA involved	Source	References
10-Epifriedelinol	428.4 µM	Bcl-xL proteins Bcl2 pathway	*Aster tataricus’* roots *Vitex penduncularis’s* leaves	(Yang et al. 2017)
Cisplatin-OA	10 µM	Bcl-2 protein LC3B-II expression	Synthetic	(Potočnjak et al. 2019)
11-oleanolic acid	43.44 µM	p16, p53, Wnt pathway, ACSL4, TfR1, FTH1, GPX4	Olive plant	(Edathara et al. 2020) (Xiaofei et al. 2021)

## Gastric cancer

Gastric cancer is common among Asian and South American populations. It has been shown to result in low survival rates. According to the Lauren classification, there are three main subtypes, but according to the WHO, there are four main subtypes. *H. pylori* and Epstein‒Barr virus are among the major causative agents of gastric cancer (Sexton et al. 2020).

The interaction between programmed cell death ligand-1 (PD-L1) and programmed cell death protein-1 helps control tumor immunity. The DNA methyltransferase 5-azacytidine regulates PD-L1 expression in breast cancer. OA at higher concentrations decreases the survival of MKN-45 cells augmented with IL-1 and attenuates PD-L1 expression. Treatment of MKN-45 cells with OA restored IL-2 levels, which indicates that T-cell activity mediates OAs’ cytotoxic effect on gastric cancer cells. OA significantly decreases demethylase activity in cells treated with IL-1 and downregulates PD-L1. OA leads to the downregulation of PD-L1 by inhibiting Tet methylcytosine dioxygenase 3 (TET3) expression. OA also inhibits the NK-kB signalling essential for attenuating PD-L1 via the demethylation of MKN-45 cells (Lu et al. 2020). Current studies showed that Treg and T helper 17 (Th17) cells are integral parts of the tumor microenvironment, and any imbalance may lead to immunosuppression in tumors. IL-6 and miRNAs regulate the Treg/Th17 imbalance in gastric cancer cells. OA also has an inhibitory effect on gastric cancer cells. qPCR results showed that in gastric cancer tissue, the miR-98-5p level decreased. In gastric cancer tissue, Treg and Th17-related factor expression levels are increased. Patients with gastric cancer have peripheral blood mononuclear cells (PBMCs) with a reduced balance of Treg/T helper 17 cells (Th17 cells). The demarcation of Treg/Th17 cells is impaired by the administration of OA, which also promotes miR-98-5p expression in the PBMCs of cancer patients. The differentiation of Treg/Th17 cells is impaired when miR-98-5p is overexpressed. miR-98-5p negatively regulates IL-6 expression. Therefore, OA regulates Treg/Th17 cell balance by regulating the expression of miR-98-5p (Xu et al. 2021). (Table 3).

**Table 3:** OA derivative and its effect on gastric cancer

Name of the derivative	IC50	Pathway/gene/RNA involved	Source	References
11-OA	> 30 µM	IL-1 protein PD-L1 expression NK-kB signaling IL-6, miR-98-5p	Olive plant	(Lu et al. 2020) (Xu et al. 2021)
HN/CDDP/OA	16.7 µM	P-gp, apoptosis pathway	Synthetic	(Li et al. 2020)

## Hepatocellular cancer

Among the different types of cancers, globally hepatocellular carcinoma (HCC) ranks as the third leading cause of death. Exposure to hepatitis B or hepatitis C and aflatoxin B1 are the major risk factors for developing hepatocellular carcinoma (Gee et al. 2018). Hepatocellular cancer treatment typically includes chemotherapy, radiofrequency ablation, etc. However, recurrence and multidrug resistance are very common, leading to the need for more efficient therapies (Li et al. 2018). Drug resistance is often caused by increased expression of P-gp, a drug transporter (Kayouka et al. 2019).

Nuclear factor erythroid 2-related factor 2 (Nrf2) which is a regulator of antioxidant and detoxifying enzymes, shows increased expression after OA treatment. The action of Nrf2 is partly mediated by farnesoid X receptor (FXR) and via NF-κB-activated pathways inhibition (Dzubak et al. 2006). Regulation of the mitochondrial pathway and X-chromosome-linked inhibitor of apoptosis protein (XIAP) promotes OA-mediated apoptosis in HuH7 cells (Shyu et al. 2010). OA modified to have azaheterocyclic groups to the A-ring shows cytotoxicity against hepatocellular cancer and BEL-7404 cell lines. The ROS/MAPK-mediated mitochondrial pathway induces apoptosis in HepG2 cells treated with a Para-aminobenzoic acid (PABA)/NO derivative of OA. Enhanced cytotoxicity is observed in HepG2 and Col-02 cells treated with OA modified with a nitro group at the C-17 position and 1-en-2-cyano-3-oxo in the A ring (Mallavadhani et al. 2013). Wang and the group demonstrated that Vascular endothelial growth factor (VEGF) and intracellular adhesion protein expression are attenuated in liver cancer after treatment with OA, which reduces viability and induces cell apoptosis. Epithelial–mesenchymal transition (EMT) is vital in tumor progression, and OA helps in inhibiting this process through the modification of inducible nitric oxide synthetase (iNOS). OA causes dimerization of iNOS, which elevates the production of NO. OA limits the ability of HCC cells to migrate and invade, which relies on the production of NO. Administration of OA reduces vimentin levels, which is a mesenchymal identifier, and elevates E-cadherin levels, which is an epithelial identifier. OA promotes the nitrification of EMT-related proteins, thus antagonizing EMT in hepatocellular cancer cells. Moreover, OA enhances antitumour activity and increases the activity of regorafenib, which is an inhibitor of angiogenesis, on vimentin, MMP2/9, and E-cadherin. Increased iNOS production enhances survival of individuals with hepatocellular cancer (Wang et al., 2019a). A recent study showed that P-gp is absent in highly invasive breast cancer cells but is present in liver cancer cells. OA decreases the viability of cells, and this effect is not impacted by verapamil, a P-gp inhibitor, in breast cancer cells. OA attenuates the survival of HepG2 cells to a greater extent in the presence of verapamil. This is due to the blocking of the efflux pump that causes OA to accumulate (Kayouka et al. 2019).

Zhou and colleagues showed how OA represses hepatocellular cancer cell growth and induces cell death and nuclear condensation in cells. OA reduces ATP levels, suggesting mitochondrial dysfunction. Bcl-2 expression is reduced, and Bax expression is increased, indicating enhanced apoptosis. P62 levels decrease, and LC3-II and Beclin-2 levels increase, indicating elevated autophagy in cells treated with OA. Akt/mTOR pathway-induced autophagy is restricted after treatment with OA. When an autophagy inhibitor is coadministered with OA, cell proliferation and apoptosis are decreased (Zhou et al. 2020b). A current study showed that essential biological pathways are regulated by miRNA gene expression, which serves as an important target for anticancer drugs. EMT is promoted by the deletion of miR-122, which leads to Hepatocellular cancer progression. OA, although not a common activator in the pathway, enhances the expression of miR-122. In addition, HepG2 cells treated with OA exhibit decreased migration and invasion rates due to increased miR-122 levels and downregulated EMT (He et al. 2021). Hosny and colleagues studied 7,12-dimethylbenz[a]anthracene (DMBA), which has tumorigenic and genotoxic effects due to the production of ROS. Few OA derivatives help inhibit DMBA-induced carcinogenesis. OA scavenges 2,2’-Azino-bis (3-ethylbenzothiazoline-6-sulfonic acid) diammonium salt (ABTS) and 2,2-Diphenyl-1-picrylhydrazyl (DPPH), indicating its antioxidant properties. It inhibits Ehrlich ascites carcinoma (EAC) and HepG2 cell line proliferation. OA increases Alanine aminotransferase (ALT), AST, proliferating cell nuclear antigen (PCNA) expression, and antioxidant levels while attenuating MDA, TNF-a, NF-kB, COX-2, and VEGF expression in mice treated with DMBA. They also showed attenuated Bcl-2 expression and elevated Caspase-3 and Beclin-1 levels. Treatment with OA improves the hepatocellular architecture (Hosny et al. 2021).

Doxorubicin (DOX) is commonly administered as an anticancer agent. DOX and OA show poor solubility in the body. This is overcome by using liposomes, which show enhanced absorption and higher drug-loading capacity. Treatment with both drugs simultaneously increased the cytotoxicity by several-fold compared to either drug alone. OA release was found to be highest in DMEM and decreased in acidic buffer. Liposome treatment shows improved cytotoxicity and anticancer effects. The number of viable cells also decreased upon treatment with the drug. DOX is most efficient in diminishing tumor growth, and DOX-encapsulated liposomes show greater cytotoxicity than OA-DOX-encapsulated liposomes. Glutathione peroxidase activity was detected only in cells treated with OA-DOX-encapsulated liposomes, indicating protection against ROS. The administration of free DOX and DOX-encapsulated liposomes resulted in the least amount of glutathione peroxidase activity. The levels of biochemical markers involved in organ toxicity increase after the administration of DOX; however, OA-DOX-encapsulated liposomes show little alteration in the expression of these markers (Sarfraz et al. 2017).

CYP450 influences some of the effects of OA, which causes the release of NO by O₂-vinyl diazenium diolates, thus exerting anticancer effects. Compound 5 (Zou et al. 2017) inhibits the proliferation of SMMC-7721 and HepG2 cells, and the highest level of NO is produced by this compound in SMMC-7721 cells; however, it does not affect normal liver cells. NO levels decrease when a CYP450 inhibitor or NO scavenger is used. The compound shows increased solubility compared to OA due to the addition of a galactosyl group to OA (Zou et al. 2017). Gee and colleagues studied how CDDO-Me affects the STAT3 pathway. CDDO-Me helps attenuate STAT3 expression, involved in the development of hepatocellular cancer. A variant of the surface marker found on the hepatitis B virus, W4P, promotes the progression of cancer via the STAT3 pathway. CDDO-Me shows cytotoxicity toward cells expressing this variant. It suppresses tumor growth, colony formation, and migration and induces the apoptosis of these cells, showing its anticancer activity. CDDO-Me suppresses the activation of STAT3 via W4P, a large surface protein Luteinizing Hormone Beta (LHB). CDDO-Me decreases cyclin D1 levels and increases p53 and p21 expression. These proteins are needed for STAT3 activation, and their alterations repress STAT3. CDDO-Me suppresses IL-6 production, which is involved in the inflammatory response; in turn, it prevents the progression of hepatocellular cancer (Gee et al. 2018). Another study showed that α-hedrin reduces cell survival and causes apoptosis through the accumulation of ROS and the depletion of GSH in hepatocellular cancer cells. It increases the levels of proapoptotic proteins and proteins associated with mitochondria-mediated apoptosis. The tumor volume and apoptosis increase with α-hedrin at higher concentrations (Li et al. 2018). Wang and group described the role of hypocrellin A (HA). HA shows anticancer activity through photodynamic effects. It can be used to increase the potency of OA as an anticancer agent. HA reduces cell density and causes cells to become rounded when administered. Yes-associated protein (YAP) and Transcription Enhancer Factor (TEA) Domain Transcription Factor (TEAD) levels and their target protein expression are attenuated after treatment with HA. Downregulation of YAP reduces hepatocellular cancer cell proliferation. HA combined with OA more strongly inhibits proliferation and migration and reduces YAP and TEAD levels in hepatocellular cancer cells (Wang et al. 2019b).

Among the different morpholide derivatives of OA, 3-succinyloxyiminoolean-12-en-28-oic acid (SMAA) has the greatest cytotoxicity, and 3-succinyloxyiminoolean-12-en-28-oic acid benzyl ester (SMAEB) has the least. 3-succinyloxyiminoolean-12-en 28-oic acid methyl ester (SMAEM) and SMAA reduce the expression of p50, while 3-succinyloxyiminoolean-12-en-28-oic acid morpholide (SMAM) and SMAEB reduce p65 levels. These proteins are involved in the NF-κB pathway. The reduction in p65 levels is correlated with SMAMs’ impact on COX-2. A reduced level of STAT5A/B in the nucleus and an increase in the nucleus are linked to the actions of SMAEB and SMAM. SMAA and SMAM increase proapoptotic protein expression, while all morpholide derivatives of OA reduce antiapoptotic protein levels (Krajka-Kuźniak et al. 2019a). Yao and colleagues studied how OA and UA affect UDP-glucuronosyltransferases (UGTs), which are metabolic enzymes involved in phase II metabolism. UGT1A1 is one of the primary UGT isoforms. Variations in these enzymes may alter drug metabolism. Transcription factors such as pregnane X receptor (PXR) and chimeric antigen receptor (CAR) regulate its expression. The UGTIA3 and UGT1A4 substrates are activated by OA and Ursolic acid (UA). Studies have been performed to understand its effect on HEPG2 cells. OA and UA upregulate the expression of UGTIA1/A3/A4/A9. OA induces PXR expression in HEPG2 cells but has no effect on CAR expression. PXR is responsible for the regulation of UGTIAs in cells treated with OA and UA. Silencing PXR reduces the induction of UGT1A1. UGT1A1 expression mediated by CAR is not influenced by OA or UA (Yao et al. 2019). Krajka-Kuźniak and colleagues demonstrated that Oleanolic acid oxime (OAO) derivatives exhibit enhanced anti-inflammatory properties due to increased hydrophilicity. Its combination with aspirin enhances its properties. The NF-κB pathway is often associated with inflammation-induced carcinogenesis. This pathway mainly regulates the IL-6 gene. COX-2 helps promote the progression and invasion of cancer cells. Therefore, its downregulation is necessary for inhibiting cancer progression. These oxime derivatives prevent the proliferation of HepG2 cells. OA conjugates with aspirin and aspirin itself show less cytotoxicity than OA and its oxime derivatives. OA derivatives, particularly morpholides, are excellent therapeutic agents. p50 and p65 levels in the NF-κB pathway decrease after treatment with OAO derivatives. The morpholide derivative of OAO conjugates with aspirin to reduce the NF-κB subunit levels to a great extent. This conjugate acts as a chemopreventive agent. A decrease in the amount of IκB kinase inhibits NF-κB pathway activation. Through the NF-κB pathway, OAO derivatives suppress the main inflammatory components, and their conjugation with aspirin enhances this effect (Krajka-Kuźniak et al., 2019b) (Fig. 3) (Table 4).

**Table 4:** OA derivative and its effect on hepatocellular cancer

Name of the derivative	IC50	Pathway/ gene/ RNA involved	Source	References
11-OA	–	Nrf2, NF-Kb, ROS/MAPK-mediated mitochondrial pathway	Olive plant	(Dzubak et al. 2006)
	> 50 µM	P-gp		(Kayouka et al. 2019)
	65 µM	vimentin, MMP2/9 and E-cadherin		(Wang et al. 2019a)
	26.91 µM	Bcl2,P62, LC3-II		(Zhou et al. 2020b)
	14.07 µM	miR-122		(He et al. 2021)
	4.25 µM	Bcl-2, LC3-II, Beclin-2, Akt/mTOR pathway, VEGF, MDA, TNF-α, NF-Kb, COX-2		(Hosny et al. 2021)
OA-DOX liposome	78.03 µM	Glutathione peroxidase activity, ROS production	Synthetic	(Sarfraz et al. 2017)
9-CDDO-Me	> 1.25 µM	IL-6 proteins STAT3 proteins	Synthetic	(Gee et al. 2018)
12-α-hedrin	18.45 µM	Mitochondrial pathway	Hedera species Nigella species	(Li et al. 2018)
13- HA	–	YAP proteins TEAD proteins	Hypocrella Bambusae Shiraia bambusicola	(Wang et al. 2019b)
14-SMAA	38 µM	NF-κB pathway	Synthetic	(Krajka-Kuźniak et al. 2019a, b)
15-SMAEB	100 µM	NF-κB pathway	Synthetic	(Krajka-Kuźniak et al. 2019a, b)
16-SMAEM	100 µM	NF-κB pathway	Synthetic	(Krajka-Kuźniak et al. 2019a, b)
17-SMAM	46 µM	NF-κB pathway	Synthetic	(Krajka-Kuźniak et al. 2019a, b)
18-Ursolic acid	–	UGTs, PXR, CAR	Apples, thyme, oregano	(Yao et al. 2019)
OAO-Asprin	> 150 µM	COX-2, IL-6, NF-kB	Synthetic	(Krajka-Kuźniak et al. 2019a, b)
CDDP/OA-LCC NPs	2 µM/35 µM	ALT, AST, mTOR pathway, NF-Kb, AMPK pathway	Synthetic	(Khan et al. 2019)

**(–)** not available

## Melanoma

Melanoma is a type of cancer associated with melanocytes that produce pigments in the skin and other areas, such as the uvea. Melanoma accounts for a small number of skin cancers but is highly associated with death. The low survival rate is due to its high metastatic activity and resistance to chemotherapeutic agents. It mostly occurs due to mutations in the BRAF V600 or neuroblastoma rat sarcoma viral oncogene homolog (N-RAS) genes. Melanoma has been treated with a variety of treatments, including radiation therapy and chemotherapy. However, these therapies tend to have side effects, and therefore, an inclination towards natural products has been observed in recent years. OA shows excellent anticancer and anti-inflammatory properties (Caunii et al. 2017).

Liu and colleagues studied that altering the sugar group linked to the aglucone of OA affects tumor progression (Liu et al. 2013). Apoptosis is induced by CDDO by increasing the amount of cytoplasmic free Ca2 + in COLO 16 cells. CDDO and CDDO-Im induce apoptosis in multidrug-resistant cells via MMP loss, the production of superoxide, the release of cytochrome c and several other factors (Hail et al. 2004). CDDO-Me and CDDO-Im promote glutathione synthesis to shield human keratinocytes from the toxicity induced by 2-chloroethyl ethyl sulfide, a sulfur mustard analog (Abel et al. 2011).

UA dose-dependently attenuates the growth of SK-MEL-2 cells at increasing concentrations. UA also affects the cell cycle progression of aneuploid and diploid cells. At lower concentrations, the diploid cells were evenly distributed across all the phases, while the aneuploidy cells were mostly concentrated at G2/M phase. S phase arrest takes place and finally the cells undergo destruction at high concentrations. The flow chamber method evaluates the adhesion of cells to certain molecules, such as Intercellular adhesion molecule 1 (ICAM-1) and vascular cell adhesion molecule 1(V-CAM 1), involved in tumor progression. Treatment with UA slightly altered the adhesion to the VCAM substrate and significantly altered the adhesion to the ICAM substrate. Compared with UA, OA has a greater impact on reducing the vascular density and the number of blood vessels in each area. Treatment with OA reduces the melanoma-associated angiogenesis reaction. Although OA is more potent as an angiogenic inhibitor than UA, its metastatic ability was not attenuated by either agent (Caunii et al. 2017).

Woo and colleagues studied how OA affects the NF-kB pathway. The survival of A375P melanoma cells was reduced with elevated concentrations of OA. OA also induced apoptosis in A375P and A375SM cells. Cleaved PARP showed greater expression in both cell lines treated with OA. Bax expression increases, and Bcl-2 expression decreases in A375SM cells, with no significant difference in expression in A375P cells. OA induces apoptosis through the regulation of p-NF-κB, an essential regulator of the NF-κB pathway. OA inhibits melanoma cell proliferation in vivo. The liver and kidneys are not affected after treatment with OA, demonstrating its nontoxic nature (Woo et al. 2021) (Table 5).

**Table 5:** OA derivative and its effect on melanoma

Name of the derivative	IC50	Pathway/gene/RNA involved	Source	References
9-CDDO-Me 1-CDDO-Im	– –	MMP	Synthetic	(Abel et al. 2011)
18-UA	58.43 µM	ICAM-1 V-CAM 1	Apples, bilberries, cranberries, elderflower, peppermint, lavender, oregano, thyme, hawthorn, and prunes	(Caunii et al. 2017)
11-OA	100 µM	NF-kB pathway, Bax, Bcl2	Olive plant	(Woo et al. 2021)
Dox@HSA-OA NPs	–	EGFR, caspase-3, caspase-7, Bcl2	Synthetic	(Kumbham et al. 2022)

**(–)** not available

## Non-small cell lung cancer

Globally non-small cell lung cancer (NSCLC) shows a prevalence of 13%. NSCLC is a commonly occurring lung cancer. Adenocarcinoma, large cell carcinoma, and squamous cell carcinoma are the most common forms of lung cancer. In recent years, immunotherapy and targeted therapy have become more and more popular for treating NSCLC (Araghi et al. 2023). VEGF expression is attenuated, and apoptosis is induced by OA in NSCLC and multidrug-resistant cells (Lucio et al. 2011). Cellular GSH is inhibited, and gamma-glutamylcysteine synthase activity decreases in cells administered with OA combined with radiation. CDDO-Me induces apoptosis via cytochrome c release and the attenuation of FLICE (FADD-like IL-1β-converting enzyme)-inhibitory protein (FLIP), which is an endogenous antagonist of caspase-8 (Zou et al. 2007). Song and colleagues studied how ROS causes damage to DNA and eventually cell death by acting on various signalling pathways. STAT3 is active in multiple forms of cancer, lung cancer included, making it a suitable target. Several OA derivatives are being used in treating NSCLC. SCZ017 is a derivative of OA that shows selective cytotoxicity toward lung cancer cells and reduces their viability. It elevates the level of apoptosis, which is induced with the help of caspase-3, and by elevating Bax/Bcl2 levels. The LC3-II/I ratio and Beclin-2 level were elevated by SZC017. These are hallmarks of autophagy. SZC017 targets the Akt pathway and decreases Akt and pAkt levels. This decreases the level of p-STAT3 in the nucleus. SZC017 leads to ROS production and the accumulation of calcium in cells. The ROS produced is inhibited with the help of NAC, which also increases the STAT3 and Akt levels and downregulates the proteins involved in autophagy. ROS regulate the apoptotic and autophagic pathways and inhibits the STA3 and Akt pathways (Song et al. 2017).

Inhibiting cancer cell autophagy reduces cell growth and helps overcome resistance to chemotherapy. Autophagy inhibitors are applied to fight cancer tumors. α-Hederin, a natural saponin, also has anticancer properties. α-Hederin upregulates LC3B-II in NSCLC, indicating an increase in the number of autophagosomes. It elevates p62 levels, demonstrating that α-Hederin is an inhibitor of autophagy and causes the accumulation of autophagosomes. The pH of lysosomes is increased in NSCLC after α-Hederin treatment, indicating the suppression of late autophagy and cathepsin D maturation in lysosomes. Paclitaxel combined with α-Hederin had greater inhibitory effects than paclitaxel alone. These compounds also increase the levels of cleaved PARP and caspase-3, indicating that caspase-centred cell death is induced in chemo-resistant cells. ROS accumulate due to the impact of α-Hederin on paclitaxel, which causes cell death (Zhan et al. 2018).

Three main groups, cyclins, their kinases, and their inhibitors, regulate the cell cycle. SSd upregulates the activity of some of these genes. c-Jun N-terminal kinase (JNK) expression increases at the sites of proliferation and apoptosis. The JNK inhibitor and SSd are combined, and their effect on cancer cells is determined. They cause cells to accumulate in the sub-G1 and sub-G2 phases, which increases apoptosis and prevents proliferation, respectively. SSd induces apoptosis via the JNK pathway. SSd and JNKi work synergistically to negatively regulate proliferation and positively regulate apoptosis. SSd alone elevates the levels of cell cycle regulator genes and JNK/pJNK activity. P53 is downregulated by JNK/pJNK, which indicates that SSd upregulates p53 levels. JNk inhibitors and SSd attenuate each other's impacts on the expression of other members (Chen et al. 2018). Wu and colleagues demonstrated the effect of SSd on NSCLC. SSd greatly inhibits the growth of NSCLCs and reduces their numbers. The initial phase of the cell cycle shows increased cell numbers and cells in G2/M decrease. SSd attenuated Cyclin D1 and CDK4 levels and elevated P27 levels. It increases the number of apoptotic cells. The levels of proliferation markers such as STAT3 are decreased, and the levels of apoptotic markers such as p44/42 and cleaved caspase-3 are elevated (Wu et al. 2020).

EGFR mutations are mostly observed in such cancers. EFGR inhibitors are often used to treat patients with this type of cancer. However, resistance to these inhibitors develops over time, paving the way for the use of OA, which shows antitumour activity against these cell lines. NO has antitumour effects and can be combined with OA to increase cytotoxicity. OA is more potent against the A549 cell line than against the other cell lines. The addition of a nitrosyl group at the end of the ester chain of OA increases its potency and enhances the spectrum of OA. The presence of an NO donor increases the activity of OA. The hybrids of OA-NO show increased cytotoxicity due to the presence of a 3-acetyl-OA (OAc-3) functional group. The inhibition of EGFR-L858R/T790M/C797S (EGFR-LTC) kinase depends on the function of the NO donor and the structure of the triterpenoid. The greater the amount of NO released, the greater the cell growth and proliferation are inhibited. OA/hederagenin and NO increase its activity against non-small cell lung cancer cell lines (Chen et al. 2019).

**Fig. 4:**
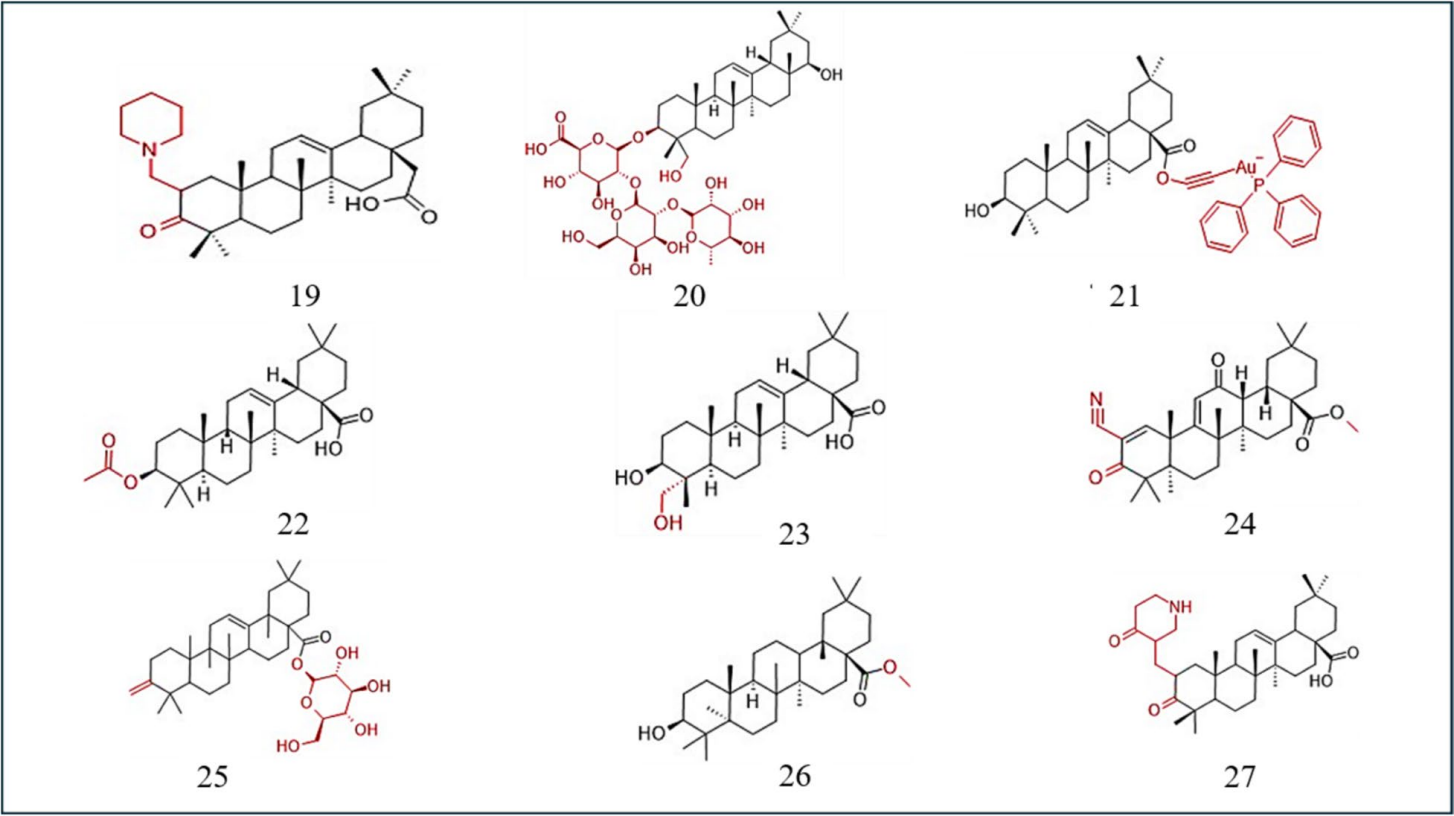
Structure of OA derivative effective against non-small cell lung, ovarian and other types of cancers. 19: SZC017; 20: Soyasaponin 1; 21: Gold-OA derivatives conjugation; 22: 3-O-acetyloleanolic acid; 23: CDDO; 24: Hederagenin; 25: Dextrose-OA; 26: OAME; 27: K73-03

Another study showed that higher doses of ursolic and OA cause a reduction in the viability of A549 cells, with UA being more cytotoxic. LC3-II levels increase with increasing incubation time, indicating that autophagy occurs. The LC3-II/LC3-1 ratio is elevated to a greater extent with uric acid. Treatment with UA and OA increases alterations in mitochondria, indicating that autophagosomes are being formed. Cells treated with UA exhibit fragmented mitochondria in autophagosomes, indicating mitophagy. UA and OA increase p62 and LC3 levels. Phosphatase and Tensin Homolog -induced kinase 1 (PINK1) and Parkin are markers of mitophagy. Both compounds elevated PINK1 expression, demonstrating that PINK1 is recruited to the mitochondrial membrane. Parkin levels increase with OA but are not affected by UA. UA induces autophagy via the AKT/mTOR pathway, while OA uses a different mechanism. Both acids stimulate ROS production. Acid-mediated inhibition of autophagy increases Nrf2 expression. Treatment of cells with OA or UA and an autophagy inhibitor significantly increased the number of cells with a low MMP, which decreased the cells' metabolic potential (Castrejón-Jiménez et al. 2019) (Fig. 4) (Table 6).

**Table 6:** OA derivative and its effect on non-small cell lung cancer

Name of the derivative	IC50	Pathway/gene/RNA involved	Source	References
9-CDDO-Me	–	FLIP, caspase-8, cytochrome c, VEGF	Synthetic	(Samudio et al. 2006, Zou et al. 2007)
19-SZC017	9.66 µM	STA3 proteins Akt proteins	Synthetic	(Song et al. 2017)
12-α-hedrin	32.43 µM	PARP proteins Caspase-3 proteins	Hedera helix	(Zhan et al. 2018)
4-SSd	–	JNK pathway	Radix Bupleuri	(Chen et al. 2018) (Wu et al. 2020)
OA/hedragenin and NO combination	–	EGFR	Synthetic	OA/hedragenin and NO
18-UA	40 µM	AKT/mTOR pathway	Apples, bilberries, cranberries, elderflower, peppermint, lavender, oregano, thyme, hawthorn, and prunes	(Castrejón-Jiménez et al. 2019)
11-OA	40 µM	PINK1/Parkin pathway	Olive plant	(Castrejón-Jiménez et al. 2019)

**(–)** not available

## Ovarian cancer

Ovarian cancer (OC) is a highly malignant type of gynecological cancer. However, due to its internal nature and multiple side effects, there are very few available therapies (Bian et al. 2020). Epithelial ovarian cancer accounts for most ovarian cancers. Ovarian cancer has a high chance of reoccurring with a low rate of survival (Jia et al. 2017). Chemotherapy based on paclitaxel and platinum, followed, or preceded by cytoreductive surgery, is the most commonly employed therapy (Sung et al. 2018).

The growth of A2780 ovarian cancer cells is attenuated by CDDO-Im via the JAK/STAT3 pathway. IL-6 secretion is reduced by CDDO-Me in paclitaxel- and cisplatin-resistant ovarian cancer cell lines and induces apoptosis via STAT3-regulated genes (Duan et al. 2009). It generates ROS in ovarian cancer cells and inhibits phosphorylated protein kinase B (p-Akt), p-mTOR, NF-Kb, p65 and antiapoptotic protein expression (Gao et al. 2011, Gao et al. 2013).

Tea saponins (TSs) have an antiproliferative effect and reduce the viability of OVCAR-3 and A2780/CP70 cells but are less cytotoxic to IOSE-364 cells. TS has a better impact than cisplatin. It inhibits colony formation and reduces the number of cells. TS increases apoptosis and causes nuclear condensation and fragmentation. Caspase-3/7 activity is increased, and pro-caspase-3 levels are decreased. TS induces apoptosis via the Bcl-2 family and not via the intrinsic apoptotic pathway. Apoptosis is induced independently of the extrinsic pathway, which relies on FAS-associated death domain protein (FADD) and death receptor 5 (DR5). VEGF expression is decreased via a hypoxia-inducible factor (HIF1-β)-independent pathway (Jia et al. 2017). Sung and colleagues reported that elevated levels of sialyl transferase and sialylated markers in tumor cells are hallmarks of cancer. Maackia amurensis agglutinin (MAA) upregulation and α2,3-linked sialylation are often observed in higher stages of cancer. Soyasaponin 1 (Ssa1) represses α2,3-linked sialylation, MAA, and Maackia amurensis lectin-2 (MAL-2) in epithelial ovarian cancer cell lines. This downregulation reduces the motility of the cells. Cell growth and multiplication are not impacted by sialylation. The cell adhesion marker cadherin (CDH) 1 was elevated, and CDH2 expression decreased. The sialyl transferase levels are manipulated by α2,3 sialylation inhibitors, demonstrating that targeting this sialylation can attenuate the migratory activity of these cells (Sung et al. 2018).

Bian and colleagues showed how OA derivatives complexed with gold affect the thioredoxin system. Cell proliferation is regulated by the thioredoxin system, which consists of thioredoxin (Trx), thioredoxin reductase (TrxR), and NADPH. Thioredoxin targeting is a promising treatment for ovarian cancer. Auranofin, a gold compound, inhibits the thioredoxin system and shows antitumour potential by interacting with the sulphur or selenium groups of TrxR. To improve the safety of metal-based treatments of ovarian cancers, they are complexed with pentacyclic triterpenes. The gold complex with an OA derivative shows the highest cytotoxicity against ovarian cancer cells. The ovarian cancer cell line showed the greatest toxicity among other cell lines. This complex inhibits TrxR, owing to its gold centre, but shows less activity than auranofin. Mitochondrial membrane potential (MMP) levels are low after treatment with the complex, indicating that mitochondrial dysfunction contributes to cell death induced by the complex. The complex induces the colocalization of ROS and endoplasmic reticulum stress (ERS) with increasing concentrations. The levels of ER chaperones and markers of ERS are elevated in the presence of the gold complex. This compound increases ROS levels, which actuates ERS, possibly leading to apoptosis. The pro-apoptotic protein expression is elevated, and the levels of pro-survival proteins are decreased. Cyclin A and cyclin-dependent kinase 2 (CDK2) levels are reduced, indicating a dependency on ROS production for the anticancer activity of the compound. Lactate dehydrogenase (LDH) is released from cells, resulting in compromised membrane integrity. NAC increases the cytotoxicity of the complex and inhibits ROS production. This complex activates ERS and produces ROS. The Trx system is necessary for maintaining redox balance, and inhibition of TrxR can offset this balance. This effect is overcome by the use of an ERS inhibitor, such as salubrinal, which inhibits ROS production. Salubrinal can also counteract the apoptotic potential of the complex. Overall, the complex inhibits tumor growth by attenuating the expression of TrxR (Bian et al. 2020) (Fig. 4) (Table 7).

**Table 7:** OA derivatives and its effect on ovarian cancer

Name of the derivative	IC50	Pathway/gene/RNA involved	Source	References
1-CDDO-Im 9-CDDO-Me	– –	p-Akt, p-mTOR, NF-Kb, p65, IL-6, JAK/STAT pathway	Synthetic	(Gao et al. 2011, Gao et al. 2013)
Tea saponins	5.9 µg/ml	VEGF,DR5,FADD	Camillia plants	(Jia et al. 2017)
20-Soyasaponin 1	–	α2,3-linked sialylation,MAA,MAL-2	*Desmodium styracifolium*	(Sung et al. 2018)
21-Gold-OA derivatives conjugation	10.24 µM	TrxR, LDH, Cyclin A, CDK2	Synthethic	Bian et al. (2020)

**(–)** not available

## Other types of cancer

### Bladder carcinoma

Novel methods are being developed for the treatment of bladder carcinoma; drug delivery systems (DDS) are also being developed. One such system is based on zeolitic imidazolate framework (ZIF) nanoparticles, which exhibit excellent properties. The cancer cell membrane (CCM) was modified to improve the targeting of the DDS. DDP is the most commonly used chemical drug; however, it can have many side effects, which can be overcome by the use of OA, which has an anticancer effect through the activation of the adenosine monophosphate-activated protein kinase (AMPK) pathway. Efficient homing can be achieved by linking Zn2 + in ZIF to phosphate groups on CCM. The hybrid nanoparticles (HNPs) of CCM-decorated ZIF with OA/DDP showed enhanced biocompatibility and drug release due to the unique ability of ZIF to respond to changes in pH. Combination therapy can achieve the same cytotoxicity at lower doses. The HP/DDP/OA group exhibited increased apoptosis, with a greater number of cells in G0/G1 phase arrest and fewer in the S phase. MDR can be reversed by OA by reducing P-gp expression. Which allows the accumulation of DDP. DDS increases the anticancer activities of OA and DDP (Chen et al. 2020).

### Colorectal cancer

3-O-acetyloleanolic acid increases the expression of colorectal cancer DR5 to cause HCT-116 cells to undergo apoptosis. (Yoo et al. 2012). The rectal cancer cell lines exhibited high sensitivity to OA, CDDO, and CDDO-Me. Cell proliferation is inhibited by OA. OA increases ROS generation in a concentration-dependent manner. NADPH oxidase (NOX) expression is elevated in HCT-15, CaCo-2, and HT-29 cells. OA silencing suppresses the ability of OA to induce ROS generation and inhibit cell proliferation. OA decreases cyclin D1 and CDK2 expression; however, this effect is suppressed by NOX silencing. OA induces increased activity of HIF-1α in CaCo-2 cells but suppresses its activity in HCT-15 and HT-29 cells. When HIF-1α is overexpressed, the inhibitory effect of OA on HCT-15 and HT-29 cell proliferation is attenuated. OA inhibits cell proliferation by downregulating HIF-1α. Suppression of HIF-1α is carried out by overexpression of NOX2 and ROS production induced by OA (Guo et al. 2017). α-Hederin is a triterpenoid saponin that brings about apoptosis in colorectal cancer cells. The survival and clonogenic ability of HCT116 and HCT8 cells decreased with increasing concentrations of α-hederin. α-Hederin induces apoptosis via the mitochondrial pathway. α-Hederin elevates LC-3 expression and increases the number of autophagosomes, indicating that it promotes autophagy in these cells. It also promotes tumorigenicity in vivo via autophagy. The Phosphorylated mammalian target of rapamycin (p-mTOR), p-ULK1, p-P70S6K, and P62 protein levels are reduced by α-hederin, while the p-AMPK and beclin-1 levels are reduced. Silencing of AMPK reversed this effect. The ROS inhibitor NAC reversed the increase in ROS production and the activation of the AMPK/mTOR pathway induced by α-hederin, indicating that NAC reverses α-hederin-induced autophagy. 3-MA, an autophagy inhibitor, inhibits α-hederin-induced autophagosome formation and decreases cell viability (Sun et al. 2019).

### Glioma and glioblastoma

These types of tumors show poor reactivity to chemotherapeutics. The proliferation of glioblastoma and neuroblastoma cells is blocked by CDDO, CDDO-Me, and CDDO-Im. The activation of caspase-3 and mitochondrial proteins by all the CDDO analogues induces apoptosis in a few paediatric solid tumor lines (Alabran et al., 2008). The growth of both U373 and human macrophages is attenuated by the suppression of CD163, IL-10, and STAT3 phosphorylation (Fujiwara et al. 2011).

### Head and neck cancer

Cisplatin is also commonly used in head and neck cancer (HNC) treatment. This type of cancer affects the upper aerodigestive tract. A combination of surgery, radiotherapy, and chemotherapy has been used to treat this type of cancer. However, MDR often develops. Antioxidant response elements (AREs) are often bound by Nrf2s’ promoter, which maintains redox homeostasis. Anticancer therapy focuses on increased ROS levels and antioxidant pathways as targets. Hederagenin, a triterpenoid, induces apoptosis via the mitochondria-mediated intrinsic pathway and helps overcome cisplatin resistance. It decreases the viability of both cisplatin-resistant and cisplatin-sensitive cells. It elevates Bax expression and decreases Bcl2 levels in cells. It activates most caspases except caspase 8. The apoptotic effect is caused by the suppression of the NF-κB pathway and ΔΨm. HED causes GSH depletion and subsequent ROS accumulation in HNC cells. It increases p53 levels in HN9-resistant and HN9-sensitive cells. HED reduced the levels of Nrf2-ARE antioxidant pathway-related proteins. It elevates Kelch-like ECH-associated protein 1 (Keap-1) levels and attenuates p62 levels. Keap- 1 knockdown causes Nrf2 elevation, which inhibits the effect of HED on HNC cells. HED inhibited the increase in Nrf2 in cisplatin-resistant cells. HED has a cytoprotective effect and does not cause harm to normal tissues. One possible target for the therapy of chemoresistant HNC cells may be the Nrf2 pathway (Kim et al. 2017).

### Hematological malignancies

Cleave poly (ADP-ribose) and augment caspase-9 and caspase-3 activity, resulting in apoptosis induced by synthetic derivatives of OA (Zhang et al. 2007). Olean-12-Eno[2,3-c] oxadiazol-28-oic acid (OEOA) causes differentiation and arrest in the G1 phase in human leukemia cells (Ng et al. 2013). Leukemia and Burkitt’s lymphoma cell lines exhibit inhibited growth when treated with oleanolic vinyl boronates (Moreira et al. 2013). CDDO has a greater effect than PPAR-gamma ligands on inducing differentiation and apoptosis via caspase activation and loss of the MMP. CDDO-Me affects NF-κB activation and gene products, which helps suppress leukemia cell proliferation. CDDO-Me and CDDO hinder the NF-kB pathway by blocking IκB kinase (IKK)-β activity (Ahmad et al. 2006).

### Osteosarcoma

Osteosarcoma is a type of malignant bone tumor that is most commonly observed among adolescents and young adults. Dextrose-OA, a derivative of OA, shows excellent anti-OS activity. By blocking the Notch pathway, OA triggers apoptosis by triggering both caspase-dependent and caspase-independent mechanisms. OA decreases cell viability and proliferation. Saos-2 cells are more strongly affected by OA than are MG63 cells. The expression of Hes-1, a target gene in the Notch pathway, was attenuated in cells treated with OA. OA suppresses Jagged1 (JAG1)-mediated apoptosis, and this effect is enhanced by the Notch inhibitor N-[N-(3,5-difluorophenacetyl)-1-alanyl]-S-phenylglycine t-butyl ester (DAPT), indicating that Notch plays a role in preventing cell proliferation. OA causes mitochondrial depolarization, which can be rescued by the addition of JAG1 ligands. OA induces a reduction in ROS, which is blocked by JAG1, the production of ROS stimulates OA-driven apoptosis and a decrease in membrane potential (Xu et al. 2018). Chen and colleagues studied how the Wnt1 signalling pathway promotes cell proliferation and invasion. Osteosarcoma progression is linked to Wnt1 signalling activation, along with elevation of c-myc, matrix metallopeptidases, and cyclin-D1, which are genes associated with cell proliferation. Wnt1 overexpression is regulated by SRY (Sex-determining Region Y)-Box (SOX9) in osteosarcoma. OA has been shown to have anti-osteosarcoma effects. OA reduces the viability, colony formation ability, and invasion of U2OS and KHOS cells. OA brings about cell death in osteosarcoma cells by promoting caspase 3 activation. OA downregulates SOX9, β-catenin, and Wnt1 expression, which can be reversed by SOX9 silencing (Chen et al. 2021).

### Prostate cancer

Prostate cancer is a hallmark of cancers among men Androgen deprivation therapy is often employed for this type of cancer. Oleanolic acid methyl ester (OAME) is a derivative of OA assessed for its antitumour potential. OAME decreases the proliferation, colony formation ability, and viability of PC-3 cells and other Prostate cancer cell lines. G2 phase arrest is seen. Phosphatidylserine is expressed on the outer membrane, and DNA is fragmented in cells treated with OAME, indicating apoptosis. OAME reduces the MMP, causes cytochrome c production, and caspase 3 activation. OAME causes ROS production in PC-3 cells. These effects brought about by OAME have also been observed in vivo (Abdelmageed et al. 2017). Treatment of prostate cancer cell lines with CDDO-Me and CDDO-Im inhibited growth due to the elevated activity of Death receptor 4 (DR4) and DR5, which serve as surfaces for TRAIL (Hyer et al. 2008). CDDO-Me also inhibits Human Telomerase Reverse Transcriptase (hTERT) expression and the Akt/NF-kB/mTOR pathway (Liu et al. 2012).

### Pancreatic cancer

Pancreatic tumors are one of the most lethal types of tumors. Several approaches have been developed to improve patient survival, with limited success. Gemcitabine (GCB) is the drug most often employed for treating pancreatic ductal adenocarcinoma (PDA). The serine protease inhibitor Kazal type-1 (SPINK1) promotes cancer progression and helps in developing resistance to the drugs employed for chemotherapy. Silencing of miR-421 results in SPINK1 overexpression. OA induces cytotoxicity and cell death in pancreatic cancer cells via a mechanism stimulated by 5-fluorouracil. CDDO-me improves cytotoxicity against cells by generating ROS, superoxide anions, and hydrogen peroxide and improves cytotoxicity against cells by generating ROS. OA induces cytotoxicity and apoptosis in pancreatic cancer cells via a mechanism mediated by 5-fluorouracil. STAT3 and IKK activity is suppressed by CDDO derivatives. CDDO-Me causes ROS production, increasing the protein repressor level of Zinc finger and Broad-Complex, Tramtrack, and Bric-a-Brac domain-containing protein 10 (ZBTB10) and attenuating the expression of cell cycle-regulating proteins (Liby et al. 2010). Treatment of AsPC-1 cells with K73-03, a novel synthetic derivative of OA, reduced their viability, proliferation rate, and colony formation ability. The expression of LC3 and Beclin-1 was upregulated in OA-treated cells, indicating the occurrence of autophagy in these cells. K73-03 increases depolarization of the mitochondrial membrane potential and increases protein degradation. It triggers the apoptosis of these cells by affecting apoptotic proteins. K73-03 upregulates MiR-421 expression in AsPC-1 cells. In pancreatic cancer cells, the SPINK1 level is low in miR-421, and K73–03 helps to reverse this effect epigenetically. Enhancers of zeste homolog 2 (EZH2) and H3K27me3 levels are attenuated in these cells. MiR-421 inhibition reverses the impact of K73-03 on SPINK1 expression. SPINK1 silencing limits cell proliferation-induced autophagy, causes mitochondrial dysfunction, reduces cell mass, reduces ATP production, and enhances the apoptotic rate. SPINK1 silencing increases the potency of K73–03-mediated inhibition of this pathway. K73-03 attenuates cell proliferation by elevating SPINK1 levels to a greater extent than GCB. Autophagy is suppressed by SPINK1 overexpression, which also causes mitochondrial dysfunction and the suppression of ATP production. In cells overexpressing SPINK1, K73-03 inhibits tumor growth and enhances apoptosis. K73-03 decreases SPINK1 expression to a greater extent than does GCB (Shopit et al. 2020).

Renal cancer has a significant death rate. An increase in cytotoxicity and resistance to drugs limits the application of chemotherapy. OA, CDDO, and CDDO-Me show high sensitivity to rectal cancer cell lines. Cell proliferation is inhibited by OA. OA increases ROS generation in a concentration-dependent manner. Nicotinamide Adenine Dinucleotide Phosphate oxidases (NOXs) expression is elevated in HCT-15, CaCo-2, and HT-29 cells. Its silencing suppresses OAs’ ability to increase ROS generation and attenuate cell growth. OA decreases cyclin D1 and CDK2 expression, however, this effect is suppressed by the silencing of NOX. OA induces increased activity of HIF-1α in CaCo-2 cells, while it suppresses its levels in HCT-15 and HT-29 cells. When HIF-1α is overexpressed, the downregulatory activity of OA on HCT-15 and HT-29 cell growth is attenuated. OA inhibits cell proliferation by downregulation of HIF-1α. Inhibition of HIF-1α is carried out by overexpression of NOX2 and ROS production brought about by OA (Guo et al. 2017). Another study shows that 2–3% of the malignancies worldwide are brought about by renal cell cancer (RCC). Treatments include partial nephrectomy and nephrectomy. SSd is a triterpenoid that displays anti-inflammatory, anti-infectious, and anti-cancer effects. EGFR is overexpressed in RCC. SSd helps suppress this activity. SSd suppresses cell viability, colony formation ability, and proliferation in 769-P and 786-O cells. SSd cause G0/G1 phase arrest and induces apoptosis in 769-P and 786-O cells. It inhibits the expression of EGFR pathway-related genes. EGFR/p38 Mitogen-activated protein kinases (MAPK) signaling pathway is suppressed by SSd to suppress cell growth (Cai et al. 2017).

### Thyroid cancer

Among the endocrine types of thyroid cancer, thyroid cancer accounts for 1–2% of systematic cancers and is the most prevalent OA that causes apoptosis via the Bax/Bcl2 pathway. Forkhead box A1 (FOXA1) is a protein found in the thyroid and is overexpressed in thyroid cancer. Thus, studies have been performed to understand how OA suppresses FOXA1 expression. OA reduces cell viability and causes nuclear condensation and fragmentation in SW579 cells. It induces apoptosis by elevating the Bax/Bcl2 levels. The cell proliferation, invasion, and clonogenic abilities of SW579 cells were reduced. OA elevates E-cadherin and suppresses vimentin. FOXA1 promotes colony formation, invasion, and migration in cells. OA elevates p-Akt levels and attenuates FOXA1 expression via the PI3K/Akt pathway (Duan et al. 2019).

### OA nanoparticle and its effect on different types of cancers

Over the past ten years, OA has advanced significantly, especially in fields like toxicity studies, chemical changes, pharmacological research, separation and purification, and therapeutic application of OA. However, the clinical uses of OA are currently very limited due to its volatility, hydrophobic nature, and low bioavailability. The biological aspect of nanotechnology has led to the development of nanoparticulate medication delivery, which could produce effective OA formulations for therapeutic use. The OA’s bioavailability and dissolving rate are improved by the nanoparticulate drug delivery system, which offers a workable formulation technique for clinical applications (Chen et al. 2011).

### Breast cancer

Recently Nanoparticles have been used for the treatment of cancer due to their unique properties, such as sustained release, greater specificity, and targeting, and improved bioavailability. OA can form nanoparticles through self-assembly, which can then be used for encapsulating drugs such as paclitaxel. Synergistic treatment is possible because OA and paclitaxel (PTX) both display anticancer effects via different mechanisms and OA, an inhibitor of P-gp, increases the concentration of PTX, which is a substrate for P-gp. Nanodrug formulation improves the antitumour efficacy of OA due to enhanced cellular uptake. When administered, PTX-OA nanoparticles decrease the size of the tumor and suppress cell proliferation (Bao et al. 2020).

### Gastric cancer

Li and the group demonstrated how cis-diamminedichloroplatinum (II) (CDDP) and OA NPs help enhance cancer treatment. CDDP is the most commonly used chemotherapeutic agent and exerts its anticancer effect by forming DNA adducts that inhibit DNA transfection. Cisplatin often causes drug resistance. Cisplatin in combination with OA helps to overcome this limitation. HNP with CDDP and OA (HN/CDDP/OA) show high bioavailability and are thus suitable as DDS. The combination of CDDP and OA reduces the dosage and enhances the anticancer effect compared to when CDDP and OA are administered alone. The combined effect of both drugs elevates apoptosis levels and promotes G0/G1 phase arrest. The incorporation of OA into a drug delivery system helps to MDR and enhances its accumulation in MGC-803 cells for improved activity. OA reverses MDR by downregulating the expression of P-gp proteins. HN/CDDP/OA shows pH-responsive release at approximately 5.5 and targets specific tumors (Li et al. 2020).

### Hepatocellular cancer

Calcium carbonate acts as a perfect delivery system due to its biocompatibility and biodegradability. Studies have investigated the effect of lipid-coated cisplatin/oleanolic acid calcium carbonate NPs (CDDP/OA-LCC NPs) on HCC cells. Both CDDP and OA reduced cell viability with increasing concentrations. A 2:35 ratio of CDDP to OA was used for efficient drug loading in the NPs. These NPs are encapsulated in a pH-sensitive carrier that selectively releases the drug. The combined NPs showed the greatest cytotoxicity. CDDP/OA-LCC NPs interact with cells efficiently and cause nuclear condensation and disintegration, characteristic of apoptotic cells. The cytotoxicity of the combined NPs was higher than that of the individual NPs. The quantity of cells in the G0/G1 and sub-G1 stages increased as a result of these NPs, with few cells in the G2/M phase, indicating increased apoptosis. The combined treatment maintained lower levels of ALT and aspartate aminotransferase (AST), indicating low hepatocellular cytotoxicity. OA shows hepatoprotective effects and counters the impact of ROS elevation. Suppression of mTOR pathway activation and the NF-κB pathway and alteration of the AMP-activated protein kinase (AMPK) pathway induces apoptosis (Khan et al. 2019).

### Melanoma

Another study showed that protein nanoparticles, such as human serum albumin nanoparticles, (HSA-NPs) have good biocompatibility, high drug loading efficiency, and avoid detection by the immune system, making them excellent carriers of anticancer agents. Human serum albumin nanoparticles interact with proteins derived from the tumor, which are acidic and have higher cysteine content and show increased activity in many cancer cells. DOX in combination with chemotherapeutic agents is used as an efficient combination therapy. HSA-NPs conjugated with OA and Dox have synergistic effects on murine melanoma and oral cancer cell lines. The amount of drug released from the HSA-NPs was greatest after 3 days, and at a pH of 5.0, administration with free Dox and Dox conjugated to HSA-NPs showed greater fluorescence signal when viewed under a fluorescence microscope after 4 h, indicating greater cellular uptake of the drug. The cell viability and IC50 values of the cell lines decreased with increasing time after treatment with HSA NP-mediated tumor-targeted Dox/OA combination therapy Human Serum Albumin Nanoparticle-mediated tumor-targeted Doxorubicin/ OA combination therapy (Dox@HSA-OA). The Annexin V assay showed that Dox@HSA-OA induced greater apoptosis than free Dox and HSA-OA in both cell lines. Both cell lines treated with Dox@HSA-OA NPs bring about G_2_/M arrest. Dox penetration was greater with Dox@HSA-OA NPs than with free Dox. Dox@HSA-OA showed greater increases in t1/2 and Cmax with reduced clearance than free Dox. Dox@HSA-OA reduced tumor size, increased DNA fragmentation and decreased the expression of the antiapoptotic marker Ki-67. Compared with cells treated with free Dox, cells treated with Dox@HSA-OA NPs showed greater upregulation of caspase-3 and caspase-7 and negative regulation of EGFR and Bcl-2 proteins (Kumbham et al. 2022) (Table 8).

**Table 8:** OA derivatives and its effect on other types of cancer

Type of cancer	IC50	Name and structure	Pathway/ gene/ RNA involved	Source	References
Bladder carcinoma	15.8 µM	HP/DDP/OA NP	P-gp	Synthetic	(Chen et al. 2020)
Colorectal cancer	–	12-α-hederin	p‑mTOR protein, p‑ULK1 protein, p‑P70S6K protein, p62 protein, mitochondrial pathway	Hedera helix	(Sun et al. 2019)
	21.85 µM	21-3-O-acetyloleanolic acid	DR5	Synthetic	(Yoo et al. 2012)
Glioma and glioblastoma	– –	22-CDDO 9-CDDO-Me	CD163, IL-10, and STAT3	Synthetic	(Fujiwara et al. 2011)
Head and neck cancer		23-Hederagenin	Mitochondria-mediated Intrinsic pathway NF-κB pathway	Chenopodium quinoa plant	(Kim et al. 2017)
Hematological malignancies	– –	24-CDDO 9-CDDO-Me	IKK-β, NF-kB	Synthetic	(Ahmad et al. 2006)
Osteosarcoma	–	25-Dextrose-OA	Notch signaling pathway	Synthetic	(Xu et al. 2018)
	–	11-OA	SOX9, β‑catenin, and Wnt1	Olive plant	(Chen et al. 2021)
Prostate	50 µM	26-OAME	Caspase 3	Leucas cephalotes	(Abdelmageed et al. 2017)
	7 µM	9-CDDO-Me	Akt/NF-kB/mTOR pathway, DR4, DR5	Synthetic	(Hyer et al. 2008)
Pancreatic	– –	11-OA 9-CDDO-Me	STAT3 and IKK	Olive plant Synthetic	(Liby et al. 2010)
	-	27-K73-03	SPINK1 gene	Synthetic	(Shopit et al. 2020)
	1.6 µM		EGFR pathway	Radix Bupleuri	(Cai et al. 2017)
Renal cancer	– –	24-CDDO 9-CDDO-Me	NADPH oxidases	Synthetic	(Guo et al. 2017)
	42.20 µmol/l	4-SSd	EGFR pathway	Radix Bupleuri	(Cai et al. 2017)
Thyroid cancer	–	11-OA	PI3K/Akt pathway,FOXA1,Bax/Bcl2	Olive plant	(Duan et al. 2019)

(–) not available

### Structure–activity relationship of OA derivatives

**Fig. 5:**
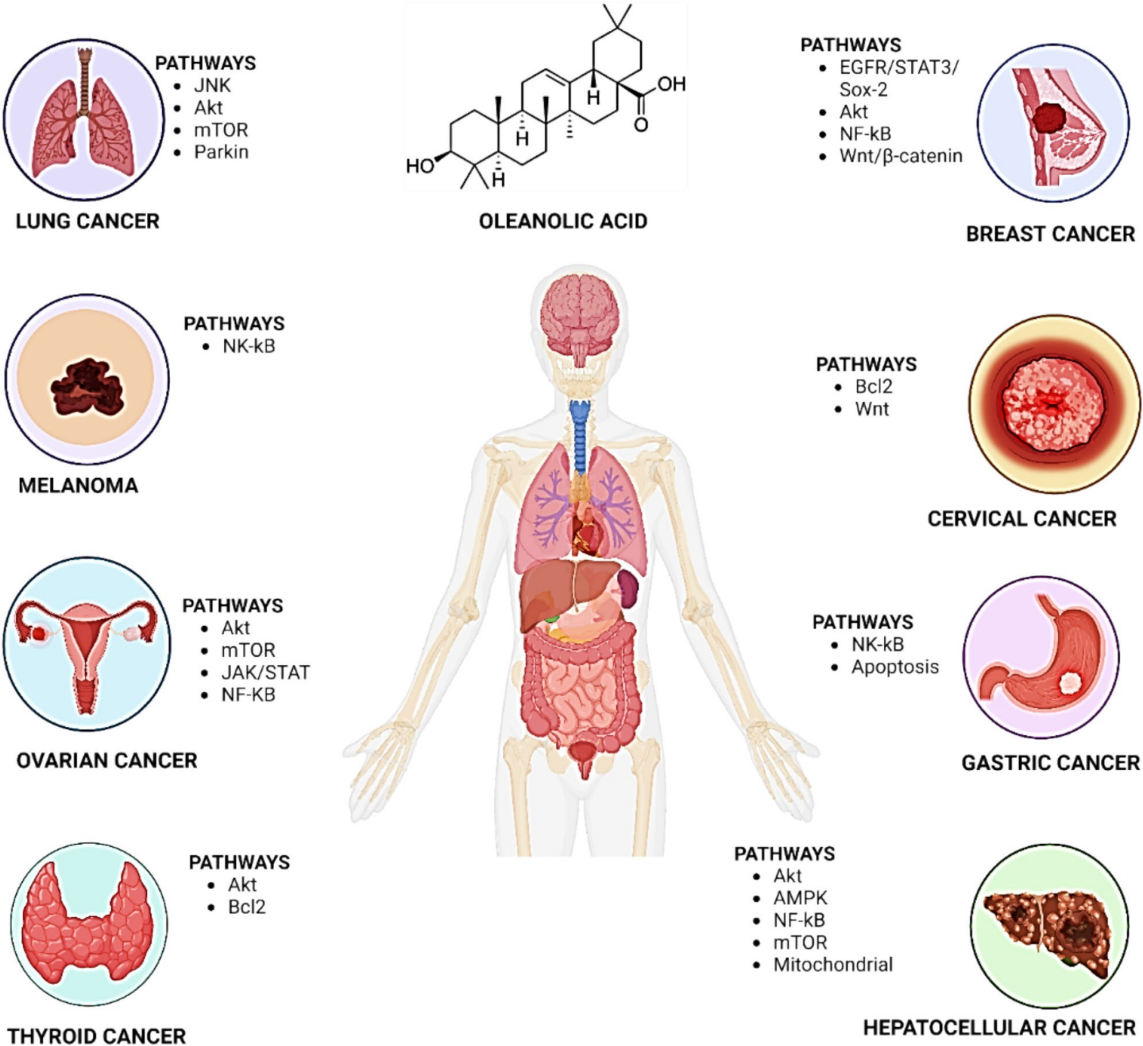
Effect of oleanolic acid and its derivatives on different cancer pathways

**Fig. 6:**
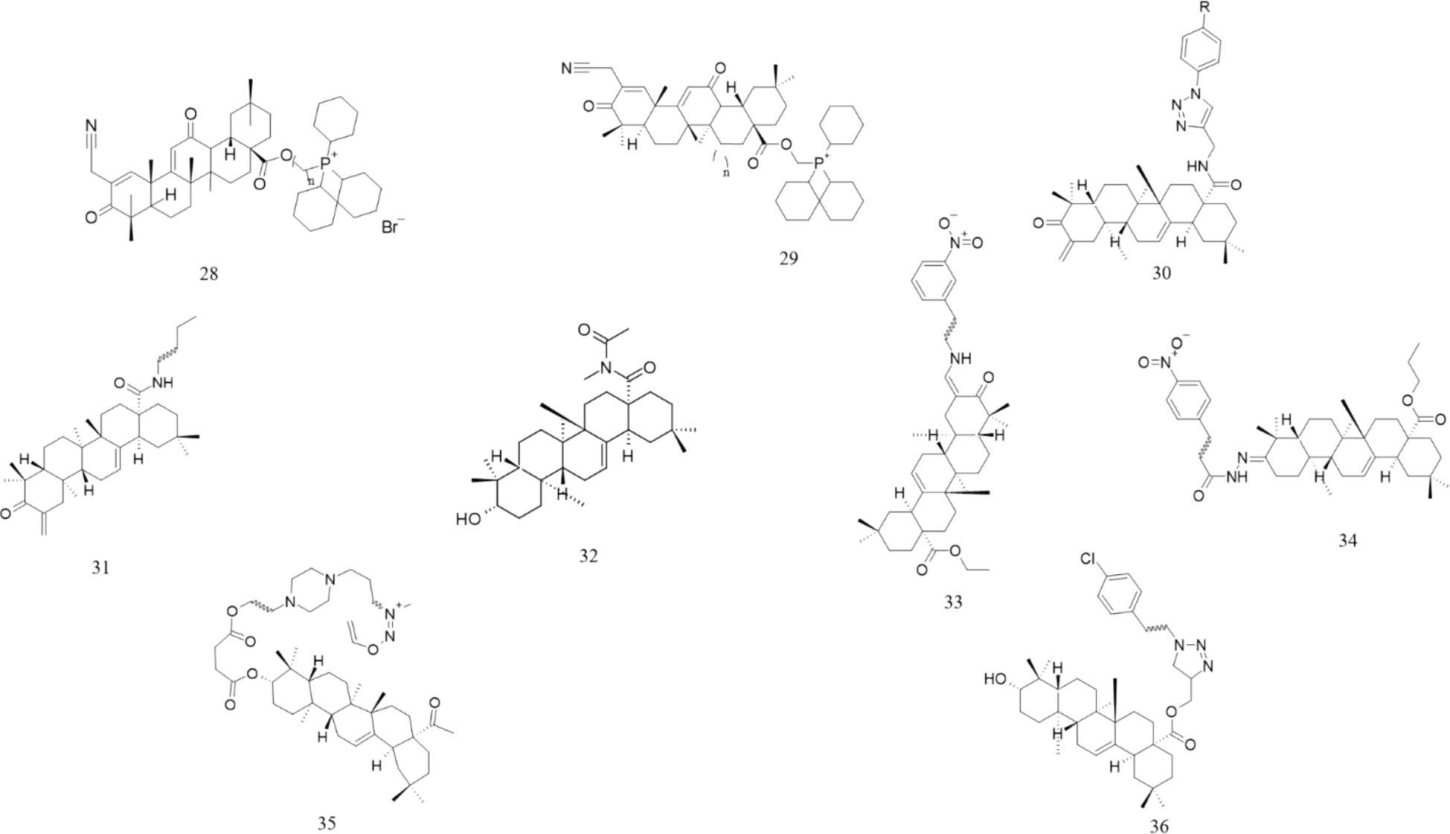
Structure–activity relationship of OA derivatives. 28, 29: Addition of TPP + or TCP + at different positions in the CDDO chain. 30, 31: Conversion of the α, β-unsaturated carbonyl derivative of OA to an amide at 28-COOH. 32: Hydroxamate derivative of OA. 33, 34: Introduction of PDGF receptor inhibitors at the C-2 or C-3 position. 35: Insertion of an O2 vinyl diazenium diolate-based group. 36: OA triazole-conjugate

The addition of triphenylphosphine cations (TPP +) or tricyclohexylphosphine cations (TCP +) (29–30 Fig. 5) along various positions of the chain of 2-cyano-3,12-dioxoolean-1,9-dien-28-oic acid (CDDO) at the 28th COOH position, improves the anticancer properties against the HCT-116, MCF-7, and A549 cell lines (Ju et al. 2021). The conversion of the α, β-unsaturated carbonyl derivative of OA to an amide at 28-COOH, had a greater antiproliferative effect on the MCF-7, HeLa, and HepG2 cell lines. Compound 30 shows greater activity against HepG2 cells, and compound 31 (Fig. 5) retards MCF-7 cell growth (Wang et al. 2020). Hydroxamate derivatives of OA have a cytotoxic impact against multiple cancer types. Compound 32 (Fig. 5) is an excellent candidate against the 518A2 cell line (Wiemann et al. 2016). Meng and his group synthesized derivatives of OA by introducing certain fragments of PDGF receptor inhibitors at the C-2 or C-3 position. Compounds 33 and 34 (Fig. 5) inhibited SGC-7901 and A549 cell lines (Meng et al. 2021). Adding a phenyl-urea moiety with a linker at the C-28 position increases its inhibitory activity against VEGFR-2 (Song et al. 2021). When a conjugated double bond is added to the C-ring of OA, the anticancer activity is enhanced against the MCF-7, HeLa, human renal adenocarcinoma (ACHN), and DU-145 cell lines. Compound 35 (Fig. 5), which is a derivative of OA, was synthesized by inserting an O2 vinyl diazenium diolate-based group that releases nitric oxide (NO) and has greater cytotoxic effects on HepG2 cells (Zou et al. 2017). The addition of a rhodamine group at the C-28 position using a piperazinyl linker to OA enhances its activity against the 518A2, A2780, HT29, MCF7, and 8505C cell lines (Yang et al. 2022). OA triazole-conjugate compound 36 (Fig. 5) shows greater anti-proliferative activity against SW480 cells than OA (Chouaib et al., 2019). Insertion of an acetyl-substituted L-arabinose moiety enhances the antiproliferative activity of OA derivatives against HL60 and A431 cells (Zhong et al. 2021). OA derivatives that are dimeric and connected at C-28 show greater activity toward multiple cell lines (Cheng et al. 2015). Parra and colleagues synthesized 264 compounds from OA and maslinic acid. These derivatives strongly affected the growth of the HT29 and B16-F10 cell lines. Several of the cytotoxic derivatives exhibited low IC50 values. The addition of a long chain ω-amino acid at the 28th carbon carboxylic group and the addition of a small acyl group at the C-3/C-2 hydroxyl group of the A-ring improve the cytotoxic effects of the derivatives (Parra et al. 2014) (Fig. 6).

### In-silico analysis of OA and its derivatives

**Fig. 7:**
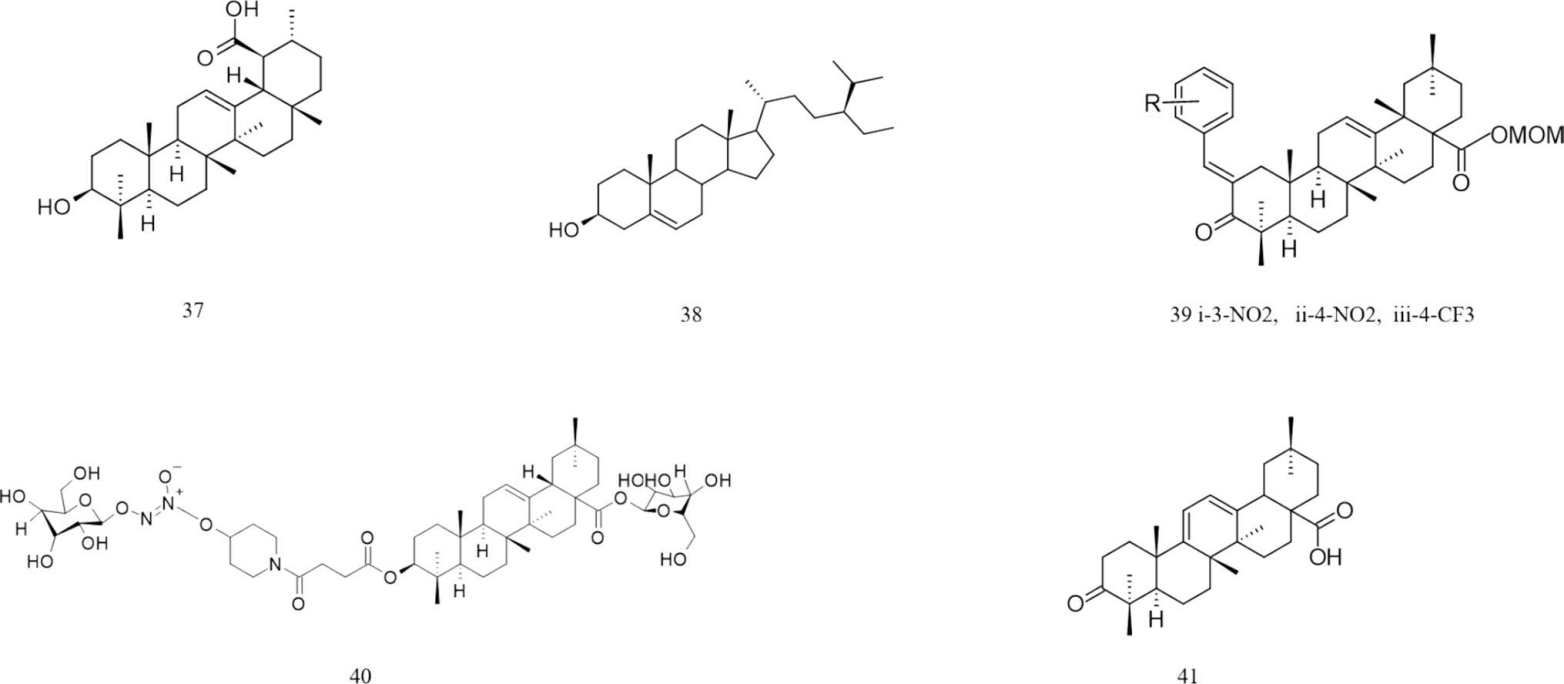
Structures of OA derivatives 37. nummularic acid, 38. β-sitosterol, 39. Derivatives of α, β‐Unsaturated Ketones Based on OA, 40. O_2_-glycosylated diazeniumdiolate OA derivative, 41. Oxy-Di-OA

To get a better understanding of molecular interactions of OA and its derivatives with various target proteins, in-silico analysis is done. Fontana and the group synthesized novel OA and Lupeol derivatives. They performed molecular docking, to determine the interaction of these derivatives with key proteins, such as NF-kB involved in leukemia progression. The derivatives showed high binding affinity for NF-kB and other related proteins. Using interactions with residues in the proteins' active site, such as hydrophobic bonds, π-π stacking, and hydrogen bonding. Modification in the derivatives structures enhanced their binding affinity, suggesting potential features critical for therapeutic efficacy, These derivatives inhibit the NF-kB pathway, impairing leukemia progression (Fontana et al. 2022). Another study showed that nummularic acid (NA) (Fig. 7 compound 37) is a plant-derived compound with strong potential as a selective inhibitor of proteins involved in the apoptotic pathways. NA forms highly stable complexes, showing binding energies of − 8.5 kcal/mol with BAX (2K7W), − 8.2 kcal/mol with BCL-2 (1K3K), − 1.9 kcal/mol with NF-κB (1NFK), and − 8.4 kcal/mol with P53 (1AIE). NA shows binding affinities and interactions similar to standard inhibitors such as doxorubicin, lapatinib, and vincristine. According to pharmacokinetic and ADME assessment, NA shows desirable physiochemical properties with great gastrointestinal absorption, moderate BBB permeability, null hepatotoxicity, and cytochrome inhibition. NA is a potential biomolecule for pharmaceutical formulations that fight cancer, with lower toxicity and greater bioavailability (Majid et al. 2022).

Molecular docking, molecular dynamic simulation (MDS), and pharmacokinetic studies were done to evaluate β-sitosterol (Fig. 7 compound 38) and OA as anti-cancer agents by inhibiting human estrogenic 17 beta-hydroxysteroid dehydrogenase type-1 (HSD17B1. Each candidate satisfied the non-toxic criterion, according to pharmacokinetic tests, molecular docking, and MDS studies showed a strong inhibitor’s interaction with HSD17B. Two essential residues in the process of enzyme inhibition are Leu149 and Pro187. Each inhibitor’s –OH and –COOH groups were essential for maintaining the inhibitor–HSD17B1 interaction. These findings show that OA and β-sitosterol are potential candidates for HSD17B1 inhibition (Kristanti et al. 2022) Senol et al. clarified that the biological activity of phenyl rings is influenced by their position and kind of substituents. Higher potency is exhibited by compounds that have a CF3 or NO2 group in the meta- or para-position of the phenyl ring. The drug-likeness parameters of the most potent compounds (Fig. 7 39i, ii, and iii), fall under Lipinski’s and Jorgensen’s rules. Molecular docking was accomplished using the induced fit docking (IFD) protocol and assessed their affinity for protein targets such as PARP1, mTOR, and P13K. Results indicated that compound Fig. 7 39i showed the greatest affinity for PARP1 (IFD score: – 10.505 kcal/mol), and compound Fig. 7 39ii displayed the greatest affinity for mTOR AND PI3K (IFD scores: –10.291 and –10.897 kcal/mol respectively) These three compound show greatest affinity and their complexes are stable with the protein targets. Molecular dynamics studies showed that the binding pocket of the target proteins showed high stability for the active compounds. These results underscore the compounds’ potential for developing inhibitors for prostate cancer (Senol et al. 2023).

*In-silico* docking studies were done to evaluate the affinity of OA with three biodegradable proteins—bovine serum albumin (BSA) (4JK4), bovine lactoferrin (1BLF), and bovine beta-lactoglobulin (3NPO), showed that OA showed highest binding affinity to BSA (− 8.2 kcal/mol), followed by beta-lactoglobulin (− 7.5 kcal/mol), and lactoferrin (− 6.2 kcal/mol). The methyl groups of OA interact with ILE297 and ARG336, by forming alkyl-alkyl bonds. OA’s cyclohexane and cyclohexene rings also showed these interactions with the ARG336, LEU304, and HIS337 residues. Albumin is the most suited protein carrier for OA among the tested options based on its strong binding affinity and energy (Shukla et al. 2023). Malleogu and the group showed that UA displays strong binding affinity and is a potential modulator of EMT-related targets. Higher fluctuations were observed for Snail-UA (Cα: 9.390 Å), Slug-UA (Cα: 5.869 Å), and Fibronectin-UA (Cα: 1.759 Å), with corresponding values for backbone, side chain, and heavy atoms reported. B-factor values also indicated structural flexibility. Root-mean-square deviation values of UA with Snail, Slug, and Fibronectin were low, indicating stable complexes. The 50 ns MDS trajectory revealed consistent hydrophobic and ionic interactions across the complexes, emphasizing UA’s strong affinity and stability in the active sites of these proteins. Hence UA can act as a potential anti-metastatic drug (Mallepogu et al. 2023).

### Patent, clinical trials, and pre-clinical trials of OA and its derivatives

Patents CN102151275A (Lin et al. 2011), CN102114022A (Lin et al. 2011), CN102114023A (Lin et al. 2011), CN102133219A (Lin et al. 2011), and CN102114021A (Lin et al. 2011) claims OAs’ application in pancreatic, colorectal, ovarian, cervical cancer, and breast cancer treatment respectively. Patent CN102838652A shows that few OA derivatives act as anti-cancer agents in liver, colon, leukemia, and oral cancers (Liu et al., 2012). OA–uridine/pyrimidine conjugates are claimed as anticancer drugs in patents CN102633855A (Cheng et al. 2012) and CN102633856A (Cheng et al. 2012), respectively. To ascertain these OA derivatives’ anticancer capabilities, they were synthesized and evaluated against a range of cancer cell lines. Patent CN102532246 A displays the role of OA aminoamide derivatives in breast cancer therapy (Shen et al. 2012). OA amidate derivatives and a number of 2-substituted OA derivatives are made and utilized pharmaceutically according to patents WO2013079018A1 and WO2013079024A1 (Xu et al. 2013). A synthesis method for OA saponin that is effective in treating tumors is claimed in patent CN103483411A (Zhu et al. 2014). Patent CN103585158A reveals a pharmaceutical composition that, at 20 μM, markedly reduced the proliferation of the rat liver cancer cell RH-35 and HepG2 cells. The composition contained OA and sulforaphane, an anticancer ingredient extracted from broccoli (Wang et al. 2014). Patent KR1020200045126 summarizes OA derivates as agents against glioblastoma (Kang-min et al. 2018). Patents CN114409725A and CNN114380884A demonstrate OA derivatives as anticancer agents (Yanqiu et al. 2022). Table 9 summarizes the patents on OA and its derivatives.

**Table 9:**
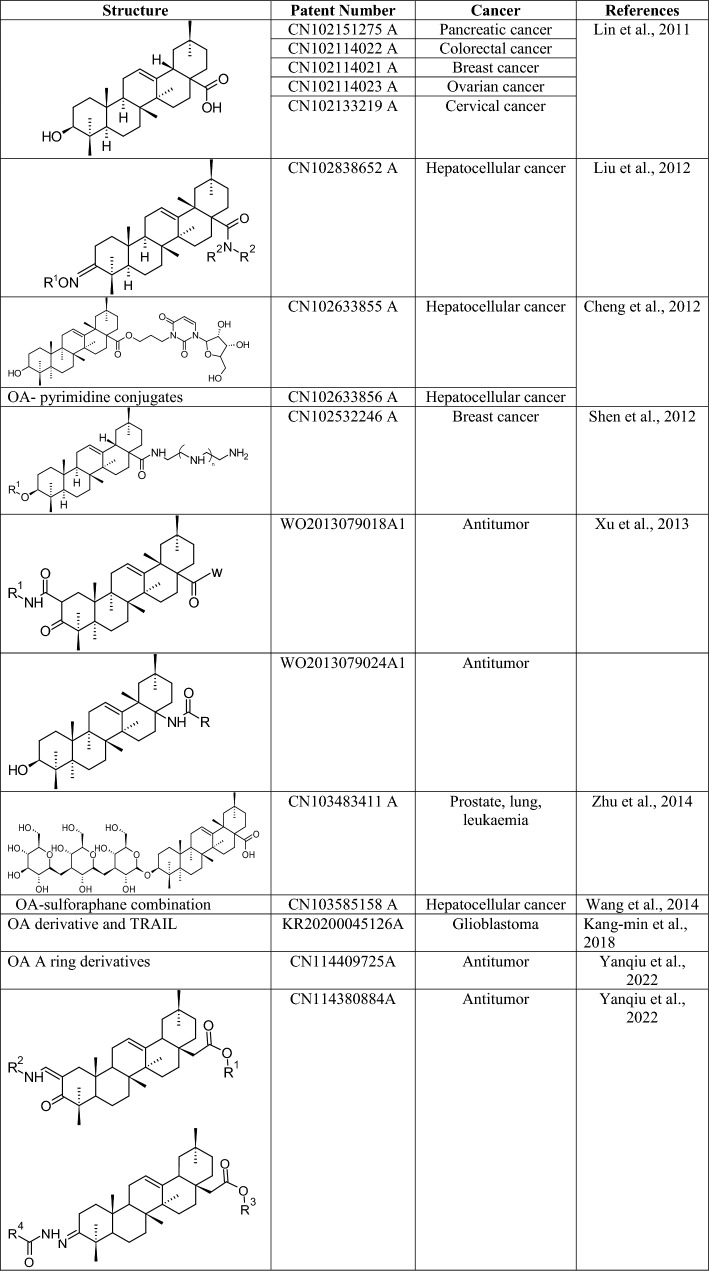
Patents on OA and its derivative

In a phase I clinical experiment, CDDO’s action in cancer cells was assessed (Speranza et al. 2012). The dose tolerance and pharmacokinetic profile were determined for a few patients. CDDO was elevated in the plasma and reached a steady state within 48 h. When administered for one year, Bardoxolone methyl showed excellent tolerance and minimal toxicity in a phase 3 trial (Hong et al. 2012). OA was mixed in the diet of C57BL/6 mice, and its effects and activity were studied. OA shows low oral bioavailability owing to abysmal gastrointestinal absorption, leading to first-pass metabolism in the liver. Formulating OA as a sodium caprate OA and freeze-dried polyvinylpyrrolidone increases its interstitial absorption and dissolution rate in Caco-2 cells. The synthesis of water-soluble amino acid analogues of OA improves its oral bioavailability. The bioavailability of OA nanosuspensions was elevated in NSCLC lines (Li et al. 2011).

### In-vivo toxicity

Huang and group studied the O2-glycosylated diazeniumdiolate OA derivative (Fig. 7 compound 40), which demonstrated anti-tumor efficacy and a promising safety profile in mice. Mice were given intravenous injections of six different doses (200, 180, 162, and 145.8 mg/kg) in an acute toxicity test, and their survival was tracked for 14 days. Only two mice survived at the maximum dose (200 mg/kg), although no deaths or aberrant behavior were seen at a low dosage (145.8 mg/kg). Compared to a similar chemical, compound 40 (Fig. 7) calculated LD50 was 173.3 mg/kg, which is substantially greater, indicating that it is a safer drug. BALB/c nude mice with subcutaneous SMMC-7721 tumors received treatment with compound 40 (Fig. 7) (3 mg/kg intravenously, three times per week for three weeks) in a different anti-HCC trial. Without changing body-weight, treatment with compound 40 (Fig. 7) dramatically decreased tumor size and weight compared to the control. These findings show that in comparison to a chemical based on furoxan, compound 40 (Fig. 7) is a powerful inhibitor of HCC tumors with reduced toxicity (Huang et al. 2012). Xiang and team investigated the acute toxicity of 3-oxours-oleana-9(11), 12-dien-28-oic acid (Oxy-Di-OA) (Fig. 7 compound 41) in male Kunming mice weighing 20 ± 2 g via intraperitoneal injection and gavage. The mice were split into six groups based on size and weight, and they were given unlimited access to water during their 12-h fast. The maximal dose of 900 mg/kg Oxy-Di-OA was produced in bean oil for the intraperitoneal injection assay. Different groups received doses of 178, 422, 600, 750, and 900 mg/kg, while one group received bean oil as a control. For the first hour, mice were monitored constantly; after that, they were monitored sporadically for 4 h, then every 24 h, and every seven days to look for any indications of toxicity or death. The LD50 was determined using the Käber method, obtaining a 714.83 mg/kg value with a 95% confidence interval of 639.73–798.73 mg/kg. At high dosages, symptoms included sluggishness and a reduction in aggression. Different doses of Oxy-Di-OA were given in the gavage experiment, whereas bean oil was given to one group as a control. The LD50 for gavage administration was higher than 2000 mg/kg, indicating minimal toxicity, and observations followed a similar timeframe. According to the findings, Oxy-Di-OA is comparatively safe at lower dosages; for additional in vivo research, 600 mg/kg is deemed innocuous (Xiang et al. 2017).

Saini and team carried out an acute toxicity study (OECD guideline 425) on female Wistar rats who were exposed to UA and OA using a metered-dose inhaler (MDI). Three male and three female rats were included in each of the two groups of rats—the control and treatment groups. For up to 14 days, the animals were monitored for clinical indications of toxicity at 1-h intervals for the first 4 h and then at 4-h intervals for the next 24 h. The treatment groups did not behave abnormally, and no fatalities or negative side effects were noted. A minor rise in blood calcium levels and variations in mean corpuscular hemoglobin concentration were the only notable alterations observed in serum biochemistry or hematology, and body and organ weights stayed constant when compared to controls. Although the lung tissue of the rats subjected to MDI exhibited congestion, hemorrhagic lesions, deformed alveolar tissue, and localized mononuclear cell infiltration, the histopathological analysis did not identify any notable changes in essential organs. Overall, the study showed that rats did not show acute toxicity to either UA or OA (Saini et al. 2022). Stepnik and group determined the potential toxicity of OA (715 µg/mL) in the zebrafish embryo using the acute toxicity test, following Organization for Economic Cooperation and Development (OECD) recommendations for chemical testing (Test No. 236) An hour after fertilization, zebrafish embryos were examined for transparency, shape, and viability. Only eggs that satisfied all requirements—namely, being round, viable, and fully transparent—were selected and transferred to 12-well plates (Sarstedt, Germany). Two groups at random—the experimental and control groups, were separated and held in each well for the whole duration of the trial, which was up to 96 h. In two batches of embryos (three wells per batch, *n* = 5–7 per well), 3000 µL of the zebrafish medium (control group) or 715 µg/mL of OA supplemented (experimental group) were used. After 23, 47, 71, and 95 h of incubation, the death rate was calculated. Incubation times of 71 and 95 h were used to measure hatchability. Following a 95-h incubation period, the following morphological defects were evaluated: posture, jaw development, eye size, hemorrhage, yolk sac necrosis, heartbeat, and heart/yolk edema. Thus, it can be said that OA at a level of 715 µg/mL is not harmful to the growing organism in vivo because it has no effect on morphology, mortality, hatchability, or muscle function (Stepnik et al. 2023).

## Discussion and future prospective

OA and its derivatives, whether derived from natural sources such as *Bupleurum falcatum* L. or synthesized chemically, have been extensively researched for their anticancer properties. This paper delves into their impact on various cancer types, with a focus on hepatocellular carcinoma (HCC) and breast cancer. In HCC, OA-DOX-encapsulated liposomes shield against reactive oxygen species (ROS), while modifications such as the addition of a galactosyl group enhance the solubility and increase the anticancer efficacy of these liposomes. Compounds such as CDDO-Me and α-hedrin exhibit promising effects by targeting inflammation, proliferation pathways, and apoptosis in HCC cells. Additionally, OA and its morpholide derivatives have the potential to suppress crucial cancer pathways, such as the NF-κB pathway, thereby impacting drug metabolism and antagonizing EMT. Similarly, in breast cancer, derivatives such as SSa, SSb, and SZC014 influence key pathways such as the JAK/STAT, Akt, and NF-κB pathways, leading to the inhibition of metastasis, angiogenesis, and cell migration, respectively. With further research being conducted on a variety of cancer types, these results point to the therapeutic potential of OA and its derivatives in the treatment of cancer.

Furthermore, these compounds hold promise beyond HCC and breast cancer, with ongoing investigations in other cancer types. By targeting multiple cellular pathways, such as the Akt/mTOR, JAK/STAT, and Wnt/β-catenin, pathways, OA, and its analogues offer a multifaceted approach to cancer therapy, potentially enhancing specificity and sensitivity to treatment. Compounds such as CDDO-Me inhibit tumor progression and survival through various mechanisms, including immune modulation and interference with chemokine signalling. Nanoformulations incorporating OA, such as OA-PTX, show potential for suppressing tumor progression by inhibiting drug efflux mechanisms. Overall, this study underscores the potential of OA and its analogues as novel therapeutic agents for cancer treatment, highlighting the importance of further research to elucidate their full therapeutic potential across diverse cancer types.

## Data Availability

Data sharing not applicable to this article as no datasets were generated or analyzed during the current study.

## Abbreviations

4-HNE: 4-Hydroxynonenal
ABTS: 2,2’-Azino-bis (3-ethylbenzothiazoline-6-sulfonic acid) diammonium salt
ACSL4: Acyl-CoA synthase long-chain family member 4
ADR: Adriamycin
AH-Me: Achyranthoside H methyl ester
ALT: Alanine aminotransferase
AMPK: Adenosine monophosphate-activated protein kinase
ARE: Antioxidant response elements
AST: Aspartate aminotransferase
Atg5: Autophagy-related gene 5
Bax: Bcl-2-associated X-protein
BCDC: *Bupleurum chinense* DC
Bcl-2: B-cell lymphoma 2
β-gal: Beta-galactosidase
BUN: Blood urea nitrogen
CAR: Chimeric antigen receptors
CCL2: Chemokine ligand 2
CCM: Cancer cell membrane
CDDO: 2-Cyano-3,12-dioxoolean-1,9-dien-28-oic acid
CDDO-Ea: 2-Cyano-3,12-dioxooleana-1,9(11)-dien-28-oic acid ethyl amide
CDDO-Im: 1-[2-Cyano-3-,12-dioxooleana-1,9(11)-dien-28-oyl] imidazole
CDDO-Me: Methyl-2-cyano-3,12-dioxooleana-1,9(11)-dien-28-oate
CDDP: Cis-diamminedichloroplatinum(II)
CDH: Cadherin
CDK2: Cyclin-dependent kinase 2
COX-2: Cyclooxygenase-2
CUK: α-Cyano-substituted α, β unsaturated ketone
CXCL16: Chemokine (C-X-C motif) ligand 16
DAPT: N-[N-(3,5-difluorophenacetyl)-1-alanyl]-S-phenylglycine t-butyl ester
DDS: Drug delivery system
DMBA: 7,12-Dimethylbenz[a]anthracene
DOX: Doxorubicin
Dox@HSA-OA: Human serum albumin nanoparticle-mediated tumor-targeted doxorubicin/ oleanolic acid combination therapy
DPPH: 2,2-Diphenyl-1-picrylhydrazyl
DR4: Death receptor 4
DR5: Death receptor 5
DRβ-H: D Rhamnose-hedrin (3b-[(a-L-arabinopyranosyl)-oxy] olean-12-en-28-oicacid)
DVL2: Dishevelled segment polarity protein 2
EAC: Ehrlich ascites carcinoma
EGFR: Epidermal growth factor receptor
EGFR^LTC^: Epidermal growth factor receptor L858R/T790M/C797S
EMT: Epithelial–mesenchymal transition
EMT-6: Experimental mammary tumour-6
ERK: Extracellular signal-regulated kinases
ERS: Endoplasmic reticulum stress
EsA: Esculentoside A
EZH2: Enhancer of zeste homolog 2
FADD: Fas-associated death domain protein
FITC: Fluorescein isothiocyanate
FLIP: FLICE (FADD-like IL-1β-converting enzyme)-inhibitory protein
FOXA1: Forkhead box A1
FTH1: Ferritin heavy chain
FXR: Farnesoid X receptor
FZD7: Frizzled class receptor 7
GEM: Gemcitabine
GPX4: Glutathione peroxidase 4
GSH: Glutathione
GSK3: Glycogen synthase kinase 3
HA: Hypocrellin A
HER2: Human epidermal growth factor receptor 2
HIF: Hypoxia inducible factors
HIMOXOL: Methyl 3-hydroxyimino-11-oxoolean-12-en28-oate
HO-1: Heme oxygenase 1
HNP: Hybrid nanoparticles
HPV: Human papilloma virus
HSA-NPs: Human serum albumin nanoparticles
HSD17B1: 17 Beta-hydroxysteroid dehydrogenase type-1
hTERT: Human telomerase reverse transcriptase
ICAM-1: Intercellular adhesion molecule 1
IFD: Induced fit docking
IKK: IκB kinase
iNOS: Inducible nitric oxide synthetase
ITGB1: Integrin beta 1
JAG1: Jagged1
JNK: C-Jun N-terminal kinase
Keap-1: Kelch-like enoyl-CoA hydratase-associated protein 1
LC3B-I/II: Light chain 3B I/II
LDH: Lactate dehydrogenase
LHB: Luteinizing hormone beta
LRP6: Low-density lipoprotein receptor-related protein 6
MAA: Maackia amurensis agglutinin
MAL-2: Maackia amurensis lectin 2
MAPK: Mitogen-activated protein kinases
MDA: Malondialdehyde
MDR1: Multi-drug resistance 1
MDS: Molecular dynamic simulation
MDI: Metered-dose inhaler
MMP: Matrix metalloproteinases
MMP: Mitochondrial membrane potential
MMP3: Matrix metallopeptidase 3
NAC: N-acetylcysteine
NF-κB: Nuclear factor kappa-light-chain-enhancer of activated B cells
NO: Nitric oxide
NOXs: Nicotinamide adenine dinucleotide phosphate oxidases
N-RAS: Neuroblastoma rat sarcoma viral oncogene homolog
NRF2: Nuclear factor erythroid 2-related factor 2
NSCLC: Non-small cell lung cancer
OA: Oleanolic acid
OAME: Oleanolic acid methyl ester
OAO: Oleanolic acid oxime
OECD: Organization for Economic Cooperation and Development
OEOA: Olean-12-eno[2,3-c] oxadiazol-28-oic acid
PABA: Para-aminobenzoic acid
p-Akt: Phosphorylated protein kinase B
PARP: Poly(adenosine diphosphate-ribose) polymerases
PBMC: Peripheral blood mononuclear cells
PCNA: Proliferating cell nuclear antigen
PCR: Polymerase chain reaction
PDA: Pancreatic ductal adenocarcinoma
PD-L1: Programmed cell death ligand 1
P-gp: P-glycoprotein
PINK1: Phosphatase and tensin homolog-induced kinase 1
p‑mTOR: Phosphorylated mammalian target of rapamycin
PTK2: Protein tyrosine kinase 2
PTX: Paclitaxel
PXN: Paxillin
PXR: Pregnane X receptor
Rh123: Rhodamine 123
RNPC: RNA-binding protein companion of MCPIP1
ROS: Reactive oxygen species
SMAA: 3-Succinyloxyiminoolean-12-en-28-oic acid
SMAEB: 3-Succinyloxyiminoolean-12-en-28-oic acid benzyl ester
SMAEM: 3-Succinyloxyiminoolean-12-en-28-oic acid methyl ester
SMAM: 3-Succinyloxyiminoolean-12-en-28-oic acid morpholide
SOD: Superoxide dismutase
SOX9: SRY (Sex-determining region Y)-box
SPINK1: Serine protease inhibitor Kazal type 1
SRB: Sulforhodamine B
SSd: Saikosaponin D
STAT1: Signal transducer and activator of transcription 1
STAT3: Signal transducer and activator of transcription 3
TAM: Tumor-associated macrophages
TEAD: Transcription enhancer factor (TEA) domain transcription factor
TET3: Tet methylcytosine dioxygenase 3
TfR1: Transferrin receptor 1
Th17 cells: T helper 17 cells
TNBC: Triple-negative breast cancer
TNF-α: Tumor necrosis factor alpha
TrxR: Thioredoxin reductase
TS: Tea saponins
UA: Ursolic acid
UGTs: UDP-glucuronosyltransferases
VASP: Vasodilator-stimulated phosphoprotein
V-CAM 1: Vascular cell adhesion molecule 1
VEGF: Vascular endothelial growth factor
Wnt: Wingless-related integration site
XIAP: X-chromosome-linked inhibitor of apoptosis protein
YAP: Yes-associated protein
ZBTB10: Zinc finger and Broad-Complex, Tramtrack, and Bric-a-Brac domain-containing protein 10
ZIF: Zeolitic imidazolate framework
Z-VAD-FMK: Carbobenzoxy-valyl-alanyl-aspartyl-[O-methyl]-fluoromethylketone

## Declarations

### Research involving human or animal participants

This article does not contain any studies with human and animal subjects performed by any of the authors.

## References

[CR1] Abdelmageed N, Morad SAF, Elghoneimy AA, Syrovets T, Simmet T et al (2017) Oleanolic acid methyl ester, a novel cytotoxic mitocan, induces cell cycle arrest and Ros-mediated cell death in castration-resistant prostate cancer PC-3 cells. Biomed Pharmacother. 10.1016/j.biopha.2017.10.027 29031200 10.1016/j.biopha.2017.10.027

[CR2] Abel EL, Bubel JD, Simper MS, Powell L, McClellan SA et al (2011) Protection against 2-chloroethyl ethyl sulfide (CEES)—induced cytotoxicity in human keratinocytes by an inducer of the glutathione detoxification pathway. Toxicol Appl Pharmacol. 10.1016/j.taap.2011.06.012 21723306 10.1016/j.taap.2011.06.012

[CR3] Ahmad R, Raina D, Meyer C, Kharbanda S, Kufe D (2006) Triterpenoid CDDO-Me blocks the NF-κB pathway by direct inhibition of IKKβ on Cys-179. J Biol Chem. 10.1074/jbc.M607160200 16998237 10.1074/jbc.M607160200

[CR4] Alabran J, Cheuk A, Liby K, Sporn M, Khan J, Letterio J, Leskov S (2008) Human neuroblastoma cells rapidly enter cell cycle arrest and apoptosis following exposure to C-28 derivatives of the synthetic triterpenoid CDDO. Cancer Biol Ther. 10.4161/cbt.7.5.5713 18277094 10.4161/cbt.7.5.5713 PMC2727860

[CR5] Araghi M, Mannani R, Heidarnejad Maleki A, Hamidi A, Rostami S et al (2023) Recent advances in non-small cell lung cancer targeted therapy: an update review. Cancer Cell Int. 10.1186/s12935-023-02990-y 37568193 10.1186/s12935-023-02990-y PMC10416536

[CR6] Bai X, Lai T, Zhou T, Li Y, Li X et al (2018) In vitro antioxidant activities of phenols and oleanolic acid from mango peel and their cytotoxic effect on A549 cell line. Molecules. 10.3390/molecules23061395 29890672 10.3390/molecules23061395 PMC6100009

[CR7] Ball MS, Bhandari R, Torres GM, Martyanov V, ElTanbouly MA et al (2020) CDDO-me alters the tumor microenvironment in estrogen receptor negative breast cancer. Sci Rep. 10.1038/s41598-020-63482-x 32300202 10.1038/s41598-020-63482-x PMC7162855

[CR8] Bao Y, Zhang S, Chen Z, Chen AT, Ma J et al (2020) Synergistic chemotherapy for breast cancer and breast cancer brain metastases via paclitaxel-loaded oleanolic acid nanoparticles. Mol Pharm. 10.1021/acs.molpharmaceut.0c00044 32150416 10.1021/acs.molpharmaceut.0c00044

[CR9] Bian M, Sun Y, Liu Y, Xu Z, Fan R et al (2020) A Gold(I) complex containing an oleanolic acid derivative as a potential anti-ovarian-cancer agent by inhibiting trxr and activating ros-mediated ERS. Chem Eur J. 10.1002/chem.202000045 32037581 10.1002/chem.202000045

[CR10] Cai C, Zhang H, Ou Y, Jiang Y, Zhong D et al (2017) Saikosaponin-D suppresses cell growth in renal cell carcinoma through EGFR/p38 signaling pathway. Neoplasma. 10.4149/neo_2017_405 28485157 10.4149/neo_2017_405

[CR11] Castrejón-Jiménez NS, Leyva-Paredes K, Baltierra-Uribe SL, Castillo-Cruz J, Campillo-Navarro M et al (2019) Ursolic and oleanolic acids induce mitophagy in A549 human lung cancer cells. Molecules. 10.3390/molecules24193444 31547522 10.3390/molecules24193444 PMC6803966

[CR12] Caunii A, Oprean C, Cristea M, Ivan A, Danciu C et al (2017) Effects of ursolic and Oleanolic on sk-mel-2 melanoma cells: in vitro and in vivo assays. Inc J Oncol. 10.3892/ijo.2017.4160 10.3892/ijo.2017.4160 PMC5673023 29039461

[CR13] Chen X, Liu C, Zhao R, Zhao P, Wu J et al (2018) Synergetic and antagonistic molecular effects mediated by the feedback loop of p53 and JNK between saikosaponin D and SP600125 on lung cancer A549 cells. Mol Pharm. 10.1021/acs.molpharmaceut.8b00595 30207732 10.1021/acs.molpharmaceut.8b00595

[CR14] Chen Z, Huang KY, Ling Y, Goto M, Duan HQ et al (2019) Discovery of an oleanolic acid/hederagenin–nitric oxide donor hybrid as an EGFR tyrosine kinase inhibitor for non-small-cell lung cancer. J Nat Prood. 10.1021/acs.jnatprod.9b00659 10.1021/acs.jnatprod.9b00659 31718182

[CR15] Chen D, Cai L, Guo Y, Chen J, Gao Q et al (2020) Cancer cell membrane-decorated zeolitic-imidazolate frameworks Codelivering cisplatin and oleanolic acid induce apoptosis and reversed multidrug resistance on bladder carcinoma cells. ACS Omega. 10.1021/acsomega.9b02261 31984255 10.1021/acsomega.9b02261 PMC6977025

[CR16] Chen X, Zhang Y, Zhang S, Wang A, Du Q et al (2021) Oleanolic acid inhibits osteosarcoma cell proliferation and invasion by suppressing the sox9/WNT1 signaling pathway. Exp Ther Med. 10.3892/etm.2021.9883 33747179 10.3892/etm.2021.9883 PMC7967867

[CR17] Cheng KG, Su CH, Yang LD, Liu J, Chen ZF (2015) Synthesis of oleanolic acid dimers linked at C-28 and evaluation of anti-tumor activity. Eur J Med Chem. 10.1016/j.ejmech.2014.10.066 25462260 10.1016/j.ejmech.2014.10.066

[CR18] Cheng L, Xia TS, Shi L, Xu L, Chen W et al (2017) D rhamnose β-hederin inhibits migration and invasion of human breast cancer cell line MDA-MB-231. Biochem. 10.1016/j.bbrc.2017.11.081 10.1016/j.bbrc.2017.11.081 29146183

[CR19] Cheng KG, Liang H, Su CH, et al. inventors; Guangxi Normal University, Peop. Rep. China. assignee. Oleanolic acid-pyrimidine conjugates, preparation method and medical application. CN102633856A. 2012.

[CR20] Cheng KG, Liang H, Su CH, et al. inventors; Guangxi Normal University, Peop. Rep. China. assignee. Preparation of oleanolic acid-uridine conjugates as antitumor agents. CN102633855A; 2012

[CR21] Chouaïb K, Romdhane A, Delemasure S, Dutartre P, Elie N, Touboul D, Ben Jannet H (2019) Regiospecific synthesis by copper- and ruthenium-catalyzed azidealkyne 1,3-dipolar cycloaddition, anticancer and anti-inflammatory activities ofoleanolic acid triazole derivatives. Arab J Chem. 10.1016/j.arabjc.2015.12.013

[CR22] Chu P, Li H, Luo R, Ahsan A, Qaed E et al (2017) Oleanolic acid derivative SZC014 inhibits cell proliferation and induces apoptosis of human breast cancer cells in a ROS-dependent way. Neoplasma. 10.4149/neo_2017_505 28592114 10.4149/neo_2017_505

[CR23] Debela DT, Muzazu SG, Heraro KD, Ndalama MT, Mesele BW et al (2021) New approaches and procedures for cancer treatment: current perspectives. SAGE Open Med. 10.1177/20503121211034366 34408877 10.1177/20503121211034366 PMC8366192

[CR24] Duan Z, Ames RY, Ryan M, Hornicek FJ, Mankin H, Seiden MV (2009) CDDO-Me, a synthetic triterpenoid, inhibits expression of IL-6 and Stat3 phosphorylation in multi-drug resistant ovarian cancer cells. Cancer Chemother Pharmacol. 10.1007/s00280-008-0785-8 18587580 10.1007/s00280-008-0785-8 PMC2875930

[CR25] Duan L, Yang Z, Jiang X, Zhang J, Guo X (2019) Oleanolic acid inhibits cell proliferation migration and invasion and induces SW579 thyroid cancer cell line apoptosis by targeting forkhead transcription factor A. Anticancer Drugs. 10.1097/cad.0000000000000777 30882397 10.1097/CAD.0000000000000777

[CR26] Dzubak P, Hajduch M, Vydra D, Hustova A, Kvasnica M, Biedermann D, Sarek J (2006) Pharmacological activities of natural triterpenoids and their therapeutic implications. Nat Prod Rep. 10.1039/B515312N 16741586 10.1039/b515312n

[CR27] Edathara PM, Chintalapally S, Makani VK, Pant C, Yerramsetty SD et al (2020) Inhibitory role of oleanolic acid and esculetin in Hela cells involve multiple signaling pathways. Gene. 10.1016/j.gene.2020.145370 33346097 10.1016/j.gene.2020.145370

[CR28] Fontana G, Badalamenti N, Bruno M, Castiglione D, Notarbartolo M, Poma P, Labbozzetta M (2022) Synthesis, in vitro and in silico analysis of new oleanolic acid and lupeol derivatives against leukemia cell lines: involvement of the NF-κB pathway. Int J Mol Sci. 10.3390/ijms23126594 35743037 10.3390/ijms23126594 PMC9223357

[CR29] Fujiwara Y, Komohara Y, Kudo R, Tsurushima K, Ohnishi K, Ikeda T, Takeya M (2011) Oleanolic acid inhibits macrophage differentiation into the M2 phenotype and glioblastoma cell proliferation by suppressing the activation of STAT3. Oncol Rep. 10.3892/or.2011.1454 21922144 10.3892/or.2011.1454

[CR30] Fukumura M, Ando H, Hirai Y, Toriizuka K, Ida Y, Kuchino Y (2009) Achyranthoside H methyl ester, a novel oleanolic acid saponin derivative from Achyranthes fauriei roots, induces apoptosis in human breast cancer MCF-7 and MDA-MB-453 cells via a caspase activation pathway. J Nat Med. 10.1007/s11418-008-0311-7 19132288 10.1007/s11418-008-0311-7

[CR31] Gamede M, Mabuza L, Ngubane P, Khathi A (2021) Preventing the onset of diabetes-induced chronic kidney disease during prediabetes: the effects of oleanolic acid on selected markers of chronic kidney disease in a diet-induced prediabetic rat model. Biomed Pharmacother. 10.1016/j.biopha.2021.111570 33932738 10.1016/j.biopha.2021.111570

[CR32] Gao X, Liu Y, Deeb D, Arbab AS, Guo AM, Dulchavsky SA, Gautam SC (2011) Synthetic oleanane triterpenoid, CDDO-Me, induces apoptosis in ovarian cancer cells by inhibiting prosurvival AKT/NF-κB/mTOR signaling. Anticancer Res. 31(11):3673–3681 22110186 PMC3711099

[CR33] Gao X, Liu Y, Deeb D, Liu P, Liu A, Arbab AS, Gautam SC (2013) ROS mediate proapoptotic and antisurvival activity of oleanane triterpenoid CDDO-Me in ovarian cancer cells. Anticancer Res. 33(1):215–221 23267148 PMC3711076

[CR34] Gee MS, Kang SB, Kim N, Choi J, Kim NJ (2018) Bardoxolone methyl suppresses hepatitis B virus large surface protein variant W4P-related carcinogenesis and hepatocellular carcinoma cell proliferation via the inhibition of signal transducer and activator of transcription 3 signaling. Pharmacology. 10.1159/000489998 29953997 10.1159/000489998

[CR35] Guimarães LP, Rocha GDG, De Queiroz RM, Martins CDA, Takiya CM et al (2017) Pomolic acid induces apoptosis and inhibits multidrug resistance protein MRP1 and migration in glioblastoma cells. Oncol Rep. 10.3892/or.2017.5895 28849227 10.3892/or.2017.5895

[CR36] Guo Y, Han B, Luo K, Ren Z, Cai L et al (2017) Nox2-Ros-HIF-1α signaling is critical for the inhibitory effect of oleanolic acid on rectal cancer cell proliferation. Biomed Pharmacother. 10.1016/j.biopha.2016.11.091 27938946 10.1016/j.biopha.2016.11.091

[CR37] Hail N, Konopleva M, Sporn M, Lotan R, Andreeff M (2004) Evidence supporting a role for calcium in apoptosis induction by the synthetic triterpenoid 2-cyano-3, 12-dioxooleana-1, 9-dien-28-oic acid (CDDO). J Biol Chem. 10.1074/jbc.M312758200 14711815 10.1074/jbc.M312758200

[CR38] Han Y, Tong Z, Wang C, Li X, Liang G (2021) Oleanolic acid exerts neuroprotective effects in subarachnoid hemorrhage rats through SIRT1-mediated HMGB1 deacetylation. Eur J Pharmacol. 10.1016/j.ejphar.2020.173811 33345851 10.1016/j.ejphar.2020.173811

[CR39] He Y, Liu X, Huang M, Wei Z, Zhang M et al (2021) Oleanolic acid inhibits the migration and invasion of hepatocellular carcinoma cells by promoting microRNA-122 expression. Pharmazie. 10.1691/ph.2021.1366 34481532 10.1691/ph.2021.1366

[CR40] Hong DS, Kurzrock R, Supko JG, He X, Naing A, Wheler J, Dezube BJ (2012) A phase I first-in-human trial of bardoxolone methyl in patients with advanced solid tumors and lymphomas. Clin Cancer Res. 10.1158/1078-0432.CCR-11-2703 22634319 10.1158/1078-0432.CCR-11-2703 PMC4494099

[CR41] Hosny S, Sahyon H, Youssef M, Negm A (2021) Oleanolic acid suppressed DMBA-induced liver carcinogenesis through induction of mitochondrial-mediated apoptosis and autophagy. Nutr Cancer. 10.1080/01635581.2020.1776887 32519911 10.1080/01635581.2020.1776887

[CR42] Huang Z, Fu J, Liu L, Sun Y, Lai Y, Ji H, Zhang Y (2012) Glycosylated diazeniumdiolate-based oleanolic acid derivatives: synthesis, in vitro and in vivo biological evaluation as anti-human hepatocellular carcinoma agents. Org Biomol Chem. 10.1039/C2OB25252J 22473516 10.1039/c2ob25252j

[CR43] Huang Z, Mou Y, Xu X, Zhao D, Lai Y et al (2017) Novel derivative of bardoxolone methyl improves safety for the treatment of diabetic nephropathy. J Med Chem. 10.1021/acs.jmedchem.7b00971 28994286 10.1021/acs.jmedchem.7b00971

[CR44] Huang M, Lu JJ, Ding J (2021) Natural products in cancer therapy: past, present and future. Nat Prod Bioprospecting. 10.1007/s13659-020-00293-7 10.1007/s13659-020-00293-7 PMC7933288 33389713

[CR45] Hyer ML, Croxton R, Krajewska M, Krajewski S, Kress CL, Lu M, Reed JC (2005) Synthetic triterpenoids cooperate with tumor necrosis factor–related apoptosis-inducing ligand to induce apoptosis of breast cancer cells. Cancer Res. 10.1158/0008-5472.CAN-04-3319 15930300 10.1158/0008-5472.CAN-04-3319

[CR46] Hyer ML, Shi R, Krajewska M, Meyer C, Lebedeva IV, Fisher PB, Reed JC (2008) Apoptotic activity and mechanism of 2-cyano-3, 12-dioxoolean-1, 9-dien-28-oic-acid and related synthetic triterpenoids in prostate cancer. Cancer Res. 10.1158/0008-5472.CAN-07-5759 18413762 10.1158/0008-5472.CAN-07-5759

[CR47] Jia LY, Wu XJ, Gao Y, Rankin GO, Pigliacampi A et al (2017) Inhibitory effects of total triterpenoid saponins isolated from the seeds of the tea plant (camellia sinensis) on human ovarian cancer cells. Molecules. 10.3390/molecules22101649 28974006 10.3390/molecules22101649 PMC6151552

[CR48] Ju W, Li N, Wang JJ, Yu NR, Lei ZC, Zhang LL, Sun JB, Chen L (2021) Design and synthesis of novel mitochondria-targeted CDDO derivatives as potential anti-cancer agents. Bioorg Chem. 10.1016/j.bioorg.2021.105249 34390971 10.1016/j.bioorg.2021.105249

[CR49] Kang X, Yang Z, Sheng J, Liu J, Xie Q et al (2017) Oleanolic acid prevents cartilage degeneration in diabetic mice via PPARƔ associated mitochondrial stabilization. Biochem Biophys Res Commun. 10.1016/j.bbrc.2017.06.127 28647370 10.1016/j.bbrc.2017.06.127

[CR50] Kang YM, Lee M, An HJ (2020) Oleanolic acid protects against mast cell-mediated allergic responses by suppressing AKT/NF-ΚB and STAT1 activation. Phytomedicine. 10.1016/j.phymed.2020.153340 33130471 10.1016/j.phymed.2020.153340

[CR51] Kang YM, Kim HM, Lee M, An HJ (2021) Oleanolic acid alleviates atopic dermatitis-like responses in vivo and in vitro. Int J Mol Sci. 10.3390/ijms222112000 34769428 10.3390/ijms222112000 PMC8584529

[CR52] Kang-Min H, Gyun N, Hee-Seon B, et al. (2018) Inventor: pharmaceutical composition for preventing or treating glioblastoma comprising oleanolic acid derivatives and TNF-related apoptosis inducing ligand as effective component. KR20200045126A.2018.

[CR53] Kayouka M, Hamade A, Saliba E, Najjar F, Landy D et al (2019) P-glycoprotein modulates oleanolic acid effects in hepatocytes cancer cells and zebrafish embryos. Chem Biol Interact. 10.1016/j.cbi.2019.108892 31704064 10.1016/j.cbi.2019.108892

[CR54] Khan MW, Zhao P, Khan A, Raza F, Raza SM et al (2019) Synergism of cisplatin-oleanolic acid co-loaded calcium carbonate nanoparticles on hepatocellular carcinoma cells for enhanced apoptosis and reduced hepatotoxicity. Int J Nanomed. 10.2147/ijn.s196651 10.2147/IJN.S196651 PMC6554709 31239661

[CR55] Kim EH, Deng CX, Sporn MB, Liby KT (2011) CDDO-imidazolide induces DNA damage, G2/M arrest and apoptosis in BRCA1-mutated breast cancer cells. J Cancer Prev. 10.1158/1940-6207.CAPR-10-0153 10.1158/1940-6207.CAPR-10-0153 PMC3076712 21372041

[CR56] Kim EH, Baek S, Shin D, Lee J, Roh JL (2017) Hederagenin induces apoptosis in cisplatin-resistant head and neck cancer cells by inhibiting the Nrf2-are antioxidant pathway. Oxid Med Cell Longev. 10.1155/2017/5498908 29456786 10.1155/2017/5498908 PMC5804377

[CR57] Krajka-Kuźniak V, Bednarczyk-Cwynar B, Narożna M, Szaefer H, Baer-Dubowska W (2019a) Morpholide derivative of the novel oleanolic oxime and succinic acid conjugate diminish the expression and activity of NF-ΚB and stats in human hepatocellular carcinoma cells. Chem Biol Interact. 10.1016/j.cbi.2019.108786 31401087 10.1016/j.cbi.2019.108786

[CR58] Krajka-Kuźniak V, Bednarczyk-Cwynar B, Paluszczak J, Szaefer H, Narożna M (2019b) Oleanolic acid oxime derivatives and their conjugates with aspirin modulate the NF-ΚB-mediated transcription in hepg2 hepatoma cells. Bioorg. 10.1016/j.bioorg.2019.103326 10.1016/j.bioorg.2019.103326 31586705

[CR59] Kristanti AN, Aminah NS, Siswanto I, Manuhara YSW, Abdjan MI, Wardana AP, Takaya Y (2022) Anticancer potential of β-sitosterol and oleanolic acid as through inhibition of human estrogenic 17beta-hydroxysteroid dehydrogenase type-1 based on an in silico approach. RSC Adv. 10.1039/D2RA03092F 35919602 10.1039/d2ra03092f PMC9278416

[CR60] Kumbham S, Paul M, Itoo A, Ghosh B, Biswas S (2022) Oleanolic acid-conjugated human serum albumin nanoparticles encapsulating doxorubicin as synergistic combination chemotherapy in oropharyngeal carcinoma and melanoma. Int J Pharm. 10.1016/j.ijpharm.2022.121479 35041911 10.1016/j.ijpharm.2022.121479

[CR61] Li W, Das S, Ng KY, Heng PW (2011) Formulation, biological and pharmacokinetic studies of sucrose ester-stabilized nanosuspensions of oleanolic acid. Pharm Res. 10.1007/s11095-011-0428-3 21479757 10.1007/s11095-011-0428-3

[CR62] Li C, Guan X, Xue H, Wang P, Wang M et al (2017) Reversal of P-glycoprotein-mediated multidrug resistance is induced by Saikosaponin D in breast cancer MCF-7/adriamycin cells. Pathol Res Pract. 10.1016/j.prp.2017.01.022 28554760 10.1016/j.prp.2017.01.022

[CR63] Li J, Wu DD, Zhang JX, Wang J, Ma JJ (2018) Mitochondrial pathway mediated by reactive oxygen species involvement in α-hederin-induced apoptosis in hepatocellular carcinoma cells. World J Gastroenterol. 10.3748/wjg.v24.i17.1901 29740205 10.3748/wjg.v24.i17.1901 PMC5937207

[CR64] Liby KT, Royce DB, Risingsong R, Williams CR, Maitra A, Hruban RH, Sporn MB (2010) Synthetic triterpenoids prolong survival in a transgenic mouse model of pancreatic cancer. Cancer Prev Res. 10.1158/1940-6207.CAPR-10-0197 10.1158/1940-6207.CAPR-10-0197 PMC2988079 20959520

[CR65] Lin XK, Liu HZ, Liu M, et al. Inventors; Lin XK. Assignee. Application of oleanolic acid in treating pancreatic cancer and its preparation. CN102151275A. 2011.

[CR66] Lin XK, Liu HZ, Liu M, et al. Inventors; Lin XK. Assignee. Application of oleanolic acid for treating colon cancer and its composition. CN102114022A. 2011.

[CR67] Lin XK, Liu HZ, Liu M, et al. Inventors; Lin XK. Assignee. Application of oleanolic acid for treating ovarian cancer and its composition. CN102114023A. 2011.

[CR68] Lin XK, Liu HZ, Liu M, et al. Inventors; Lin XK. Assignee. New application and pharmaceutical preparation of oleanolic acid for treating breast carcinoma. CN102114021A. 2011.

[CR69] Lisiak N, Paszel-Jaworska A, Totoń E, Rubiś B, Pakuła M et al (2017) Semisynthetic oleanane triterpenoids inhibit migration and invasion of human breast cancer cells through downregulated expression of the ITGB1/ptk2/PXN pathway. Chem Bio Interact. 10.1016/j.cbi.2017.03.008 10.1016/j.cbi.2017.03.008 28322779

[CR70] Lisiak NM, Lewicka I, Kaczmarek M, Kujawski J, Bednarczyk-Cwynar B (2021) Oleanolic acid’s semisynthetic derivatives HIMOXOL and BR-HIMOLID show proautophagic potential and inhibit migration of HER2-positive breast cancer cells in vitro. Int J Mol Sci. 10.3390/ijms222011273 34681931 10.3390/ijms222011273 PMC8538366

[CR71] Liu Y, Gao X, Deeb D, Gautam SC (2012b) Oleanane triterpenoid CDDO-Me inhibits Akt activity without affecting PDK1 kinase or PP2A phosphatase activity in cancer cells. Biochem Biophys Res Commun. 10.1016/j.bbrc.2011.12.007 22177954 10.1016/j.bbrc.2011.12.007 PMC3264055

[CR72] Liu Q, Liu H, Zhang L, Guo T, Wang P, Geng M, Li Y (2013) Synthesis and antitumor activities of naturally occurring oleanolic acid triterpenoid saponins and their derivatives. Eur J Med Chem. 10.1016/j.ejmech.2013.04.016 23639650 10.1016/j.ejmech.2013.04.016

[CR73] Liu C, Dong L, Sun Z, Wang L, Wang Q et al (2018a) Esculentoside a suppresses breast cancer stem cell growth through STEMNESS attenuation and apoptosis induction by blocking IL-6/Stat3 Signaling pathway. Phytother Res. 10.1002/ptr.6172 30080291 10.1002/ptr.6172

[CR74] Liu Y, Gao L, Zhao X, Guo S, Liu Y et al (2018b) Saikosaponin a protects from pressure overload-induced cardiac fibrosis via inhibiting fibroblast activation or endothelial cell EndMT. Int J Biol Sci. 10.7150/ijbs.27022 30443195 10.7150/ijbs.27022 PMC6231222

[CR75] Liu Y, Liu DK, Xie XJ, et al. Inventors; Tianjin Institute of Pharmaceutical Research, Peop. Rep. China. assignee. An oleanolic acid derivative, its preparation method and application as antitumor agents. CN102838652A. 2012.

[CR76] Lixing X, Zhouye J, Liting G, Ruyi Z, Rong Q et al (2017) Saikosaponin- D -mediated downregulation of neurogenesis results in cognitive dysfunction by inhibiting AKT/foxg-1 pathway in mice. Toxicol Lett. 10.1016/j.toxlet.2017.11.009 29129800 10.1016/j.toxlet.2017.11.009

[CR77] Lu X, Li Y, Yang W, Tao M, Dai Y et al (2020) Inhibition of NF-ΚB is required for oleanolic acid to downregulate PD-L1 by promoting DNA demethylation in gastric cancer cells. J Biochem. 10.1002/jbt.22621 32894642 10.1002/jbt.22621

[CR78] Lúcio KA, Rocha GDG, Monção-Ribeiro LC, Fernandes J, Takiya CM, Gattass CR (2011) Oleanolic acid initiates apoptosis in non-small cell lung cancer cell lines and reduces metastasis of a B16F10 melanoma model in vivo. PLoS ONE. 10.1371/journal.pone.0028596 22174843 10.1371/journal.pone.0028596 PMC3235133

[CR79] Łukasiewicz S, Czeczelewski M, Forma A, Baj J, Sitarz R et al (2021) Breast cancer—epidemiology, risk factors, classification, prognostic markers, and current treatment strategies—an updated review. Cancers. 10.3390/cancers13174287 34503097 10.3390/cancers13174287 PMC8428369

[CR80] Ma Q, Ga F, He X, Li K, Gao Y et al (2019) Antitumor effects of saikosaponin B2 on breast cancer cell proliferation and migration. Mol Med Rep. 10.3892/mmr.2019.10385 31257464 10.3892/mmr.2019.10385

[CR81] Majid M, Farhan A, Asad MI, Khan MR, Hassan SSU, Haq IU, Bungau S (2022) An extensive pharmacological evaluation of new anti-cancer triterpenoid (nummularic acid) from Ipomoea batatas through in vitro, in silico, and in vivo studies. Molecules. 10.3390/molecules27082474 35458672 10.3390/molecules27082474 PMC9030838

[CR82] Mallavadhani UV, Mahapatra A, Pattnaik B, Vanga N, Suri N, Saxena AK (2013) Synthesis and anti-cancer activity of some novel C-17 analogs of ursolic and oleanolic acids. Med Chem Res. 10.1007/s00044-012-0106-y

[CR83] Mallepogu V, Sankaran KR, Pasala C, Bandi LR, Maram R, Amineni UM, Meriga B (2023) Ursolic acid regulates key EMT transcription factors, induces cell cycle arrest and apoptosis in MDA-MB-231 and MCF-7 breast cancer cells, an in-vitro and in silico studies. J Cell Biochem. 10.1002/jcb.30496 37992132 10.1002/jcb.30496

[CR84] Chen M, Zhong Z, Tan W, Wang S, Wang Y (2011) Recent advances in nanoparticle formulation of oleanolic acid. Chin Med. 10.1186/1749-8546-6-20 21619582 10.1186/1749-8546-6-20 PMC3123256

[CR85] Meng YQ, Zhou Y, Li QW, Tong SM, Kuai ZY, Li XX (2021) Synthesis of oleanolic acid analogues targeting PDGF receptor inhibitors and their antitumor biological activities. J Asian Nat Prod Res. 10.1080/10286020.2020.1717476 32102552 10.1080/10286020.2020.1717476

[CR86] Moreira VM, Salvador JA, Simões S, Destro F, Gavioli R (2013) Novel oleanolic vinyl boronates: synthesis and antitumor activity. Eur J Med Chem. 10.1016/j.ejmech.2013.01.040 23455056 10.1016/j.ejmech.2013.01.040

[CR87] Ng YP, Chen Y, Hu Y, Ip FC, Ip NY (2013) Olean-12-eno [2, 3-c][1, 2, 5] oxadiazol-28-oic acid (OEOA) induces G1 cell cycle arrest and differentiation in human leukemia cell lines. PLoS ONE. 10.1371/journal.pone.0063580 23696836 10.1371/journal.pone.0063580 PMC3656051

[CR88] Parra A, Martin-Fonseca S, Rivas F, Reyes-Zurita FJ, Medina-O’Donnell M et al (2014) Solid-phase library synthesis of bi-functional derivatives of oleanolic and maslinic acids and their cytotoxicity on three cancer cell lines. ACS Comb Sci. 10.1021/co500051z 24916186 10.1021/co500051z

[CR89] Pattnaik B, Lakshma Nayak V, Ramakrishna S, Venkata Mallavadhani U (2016) Synthesis of ring-C modified oleanolic acid derivatives and their cytotoxicevaluation. Bioorg Chem. 10.1016/j.bioorg.2016.08.001 27522460 10.1016/j.bioorg.2016.08.001

[CR90] Potočnjak I, Šimić L, Vukelić I, Domitrović R (2019) Oleanolic acid attenuates cisplatin-induced nephrotoxicity in mice and chemosensitizes human cervical cancer cells to cisplatin cytotoxicity. Food Chem Toxicol. 10.1016/j.fct.2019.110676 31306688 10.1016/j.fct.2019.110676

[CR91] Saini V, Debnath SK, Maske P, Dighe V, Srivastava R (2022) Targeted delivery of ursolic acid and oleanolic acid to lungs in the form of an inhaler for the management of tuberculosis: pharmacokinetic and toxicity assessment. PLoS ONE. 10.1371/journal.pone.0278103 36580459 10.1371/journal.pone.0278103 PMC9799288

[CR92] Samudio I, Konopleva M, Pelicano H, Huang P, Frolova O, Bornmann W, Andreeff M (2006) A novel mechanism of action of methyl-2-cyano-3, 12 dioxoolean-1, 9 diene-28-oate: direct permeabilization of the inner mitochondrial membrane to inhibit electron transport and induce apoptosis. Mol Pharmacol. 10.1124/mol.105.018051 16410408 10.1124/mol.105.018051

[CR93] Sarfraz M, Afzal A, Raza SM, Bashir S, Madni A et al (2017) Liposomal co-delivered oleanolic acid attenuates doxorubicin-induced multi-organ toxicity in hepatocellular carcinoma. Oncotarget. 10.18632/oncotarget.17559 28525367 10.18632/oncotarget.17559 PMC5564550

[CR94] Şenol H, Ghaffari-Moghaddam M, Bulut Ş, Akbaş F, Köse A, Topçu G (2023) Synthesis and anticancer activity of novel derivatives of α, β-unsaturated ketones based on oleanolic acid: in vitro and in silico studies against prostate cancer cells. Chem Biodivers. 10.1002/cbdv.202301089 37596247 10.1002/cbdv.202301089

[CR95] Sexton RE, Al Hallak MN, Diab M, Azmi AS (2020) Gastric cancer: a comprehensive review of current and future treatment strategies. Cancer Metastasis Rev. 10.1007/s10555-020-09925-3 32894370 10.1007/s10555-020-09925-3 PMC7680370

[CR96] Shamsee ZR, Al-Saffar AZ, Al-Shanon AF, Al-Obaidi JR (2018) Cytotoxic and cell cycle arrest induction of pentacyclic triterpenoides separated from Lantana Camara leaves against MCF-7 cell line in vitro. Mol Biol Rep. 10.1007/s11033-018-4482-3 30426385 10.1007/s11033-018-4482-3

[CR97] Shen YQ, Shi DF, Tang JB, et al. Inventors; Zhejiang University, Peop. Rep. China. assignee. Oleanolic acid derivatives, preparation method and application. CN102532246A. 2012.

[CR98] Shopit A, Li X, Tang Z, Awsh M, Shobet L et al (2020) Mir-421 up-regulation by the oleanolic acid derivative K73–03 regulates epigenetically SPINK1 transcription in pancreatic cancer cells leading to metabolic changes and enhanced apoptosis. Pharmacol Res. 10.1016/j.phrs.2020.105130 32818653 10.1016/j.phrs.2020.105130

[CR99] Shukla VN, Mehata AK, Setia A, Kumari P, Mahto SK, Muthu MS, Mishra SK (2023) EGFR targeted albumin nanoparticles of oleanolic acid: In silico screening of nanocarrier, cytotoxicity and pharmacokinetics for lung cancer therapy. Int J Biol Macromol. 10.1016/j.ijbiomac.2023.125719 37419266 10.1016/j.ijbiomac.2023.125719

[CR100] Shyu MH, Kao TC, Yen GC (2010) Oleanolic acid and ursolic acid induce apoptosis in HuH7 human hepatocellular carcinoma cells through a mitochondrial-dependent pathway and downregulation of XIAP. J Agric Food Chem. 10.1021/jf100574j 20415421 10.1021/jf100574j

[CR101] So JY, Wahler JE, Yoon T, Smolarek AK, Lin Y, Shih WJ, Suh N (2013) Oral administration of a gemini vitamin D analog, a synthetic triterpenoid and the combination prevents mammary tumorigenesis driven by ErbB2 overexpression. Cancer Prev Res. 10.1158/1940-6207.CAPR-13-0087 10.1158/1940-6207.CAPR-13-0087 PMC3767182 23856074

[CR102] Song Y, Kong L, Sun B, Gao L, Chu P et al (2017) Induction of autophagy by an oleanolic acid derivative, Szc017, promotes ros-dependent apoptosis through Akt and JAK2/STAT3 signaling pathway in human lung cancer cells. Cell Bio Int. 10.1002/cbin.10868 10.1002/cbin.10868 28880428

[CR103] Song YL, Zhang PB, Tong RJ, Li L, Meng YQ (2021) Design and synthesis of VEGFR-2 inhibitors based on oleanolic acid moiety. J Asian Nat Prod Res. 10.1080/10286020.2019.1706500 31888388 10.1080/10286020.2019.1706500

[CR104] Speranza G, Gutierrez ME, Kummar S, Strong JM, Parker RJ, Collins J, Chen A (2012) Phase I study of the synthetic triterpenoid, 2-cyano-3, 12-dioxoolean-1, 9-dien-28-oic acid (CDDO), in advanced solid tumors. Cancer Chemother Pharmacol. 10.1007/s00280-011-1712-y 21805353 10.1007/s00280-011-1712-y PMC4490274

[CR105] Stępnik K, Kukula-Koch W, Plazinski W, Rybicka M, Gawel K (2023) Neuroprotective properties of oleanolic acid—computational-driven molecular research combined with in vitro and in vivo experiments. Pharmaceuticals. 10.3390/ph16091234 37765042 10.3390/ph16091234 PMC10536188

[CR106] Sun J, Feng Y, Wang Y, Ji Q, Cai G et al (2019) Α-hederin induces autophagic cell death in colorectal cancer cells through reactive oxygen species dependent AMPK/mTOR signaling pathway activation. Int J Oncol. 10.3892/ijo.2019.4757 30896843 10.3892/ijo.2019.4757 PMC6438428

[CR107] Sung PL, Wen KC, Horng HC, Chang CM, Chen YJ (2018) The role of α2,3-linked sialylation on clear cell type epithelial ovarian cancer. Taiwan J Obstet Gynecol. 10.1016/j.tjog.2018.02.015 29673670 10.1016/j.tjog.2018.02.015

[CR108] Wang J, Qi H, Zhang X, Si W, Xu F, Hou T, Zhou H, Wang A, Li G, Liu Y, Fang Y, Piao H, Liang X (2018) Saikosaponin D from radix Bupleuri suppresses triple-negative breast cancer cell growth by targeting β-catenin signaling. Biomed Pharmacother. 10.1016/j.biopha.2018.09.038 30248540 10.1016/j.biopha.2018.09.038

[CR109] Wang H, Zhong W, Zhao J, Zhang H, Zhang Q et al (2019a) Oleanolic acid inhibits epithelial–mesenchymal transition of hepatocellular carcinoma by promoting inos dimerization. Mol Cancer Ther. 10.1158/1535-7163.mct-18-0448 30297361 10.1158/1535-7163.MCT-18-0448

[CR110] Wang L, Wang J, Cao Y, Li W, Wang Y et al (2019b) Molecular evidence for better efficacy of hypocrellin A and oleanolic acid combination in suppression of HCC growth. Eur J Pharmacol. 10.1016/j.ejphar.2018.10.042 30391347 10.1016/j.ejphar.2018.10.042

[CR111] Wang W, Wu L, Li J, Ji J, Chen K et al (2019c) Alleviation of hepatic ischemia reperfusion injury by oleanolic acid pretreating via reducing HMGB1 release and inhibiting apoptosis and autophagy. Mediators Infamm. 10.1155/2019/3240713 10.1155/2019/3240713 PMC6604292 31316298

[CR112] Wang SS, Zhang QL, Chu P, Kong LQ, Li GZ, Li YQ, Yang L, Zhao WJ, Guo XH, Tang ZY (2020) Synthesis and antitumor activity of α, β-unsaturated carbonyl moiety- containing oleanolic acid derivatives targeting PI3K/AKT/mTORsignaling pathway. Bioorg Chem. 10.1016/j.bioorg.2020.104036 32629283 10.1016/j.bioorg.2020.104036

[CR113] Wang X, Hai CX. Inventors; Fourth Military Medical University, PLA, Peop. Rep. China. Assignee. Pharmaceutical composition and its application in preparation of drugs for treating liver cancer. CN103585158A. 2014.

[CR114] Wiemann J, Heller L, Csuk R (2016) Targeting cancer cells with oleanolic and ursolic acid derived hydroxamates. Bioorg Med Chem Lett. 10.1016/j.bmcl.2015.12.064 26750249 10.1016/j.bmcl.2015.12.064

[CR115] Woo JS, Yoo ES, Kim SH, Lee JH, Han SH et al (2021) Anticancer effects of oleanolic acid on human melanoma cells. Chem Biol Interact. 10.1016/j.cbi.2021.109619 34364837 10.1016/j.cbi.2021.109619

[CR116] Wu S, Chen W, Liu K, Ren F, Zheng D et al (2020) Saikosaponin d inhibits proliferation and induces apoptosis of non-small cell lung cancer cells by inhibiting the STAT3 pathway. J Int Med Res. 10.1177/0300060520937163 32962498 10.1177/0300060520937163 PMC7780581

[CR117] Xiang H, Han Y, Zhang Y, Yan W, Xu B, Chu F, Lei H (2017) A new oleanolic acid derivative against CCl4-induced hepatic fibrosis in rats. Int J Mol Sci. 10.3390/ijms18030553 28272302 10.3390/ijms18030553 PMC5372569

[CR118] Xiaofei J, Mingqing S, Miao S, Yizhen Y, Shuang Z et al (2021) Oleanolic acid inhibits cervical cancer hela cell proliferation through modulation of the ACSL4 Ferroptosis Signaling pathway. Biochem. 10.1016/j.bbrc.2021.01.028 10.1016/j.bbrc.2021.01.028 33548628

[CR119] Xu Y, Shu B, Tian Y, Wang G, Wang Y et al (2018) Oleanolic acid induces osteosarcoma cell apoptosis by inhibition of Notch signaling. Mol Carcinog. 10.1002/mc.22810 29566282 10.1002/mc.22810

[CR120] Xu QF, Peng HP, Lu XR, Hu Y, Xu ZH et al (2021) Oleanolic acid regulates the TREG/th17 imbalance in gastric cancer by targeting IL-6 with mir-98-5p. Cytokine. 10.1016/j.cyto.2021.155656 34388475 10.1016/j.cyto.2021.155656

[CR121] Xu RZ, Rong FG, Lai HX, et al. inventors; Hangzhou Bensheng Pharmaceutical Co. Ltd., Peop. Rep. China. assignee. 2-Substituted oleanolic acid deriv. useful in treatment of cancer and its preparation. WO2013079018A1. 2013.

[CR122] Xu RZ, Rong FG, Lai HX, et al. inventors; Hangzhou Bensheng Pharmaceutical Co. Ltd., Peop. Rep. China. assignee. Oleanolic acid amidate derivatives useful in treatment of cancer and their preparation. WO2013079024A1. 2013.

[CR123] Yang J, Liao D, Chen C, Liu Y, Chuang TH, Xiang R, Luo Y (2013) Tumor-associated macrophages regulate murine breast cancer stem cells through a novel paracrine EGFR/Stat3/Sox-2 signaling pathway. Stem Cells. 10.1002/stem.1281 23169551 10.1002/stem.1281

[CR124] Yang J, Fa J, Li B (2017) Apoptosis induction of epifriedelinol on human cervical cancer cell line. Afr J Tradit Complement Altern Med. 10.21010/ajtcam.v14i4.10 28638870 10.21010/ajtcam.v14i4.10 PMC5471486

[CR125] Yang YH, Dai SY, Deng FH, Peng LH, Li C, Pei YH (2022) Recent advances in medicinal chemistry of oleanolic acid derivatives. Phytochemistry. 10.1016/j.phytochem.2022.113397 36029846 10.1016/j.phytochem.2022.113397

[CR126] Yanqiu M, Yanling S, Wong, et al, inventor: Shenyang University of Chemical Technology, assignee. Oleanolic acid A ring derivative with anti-tumor activity and preparation method thereof. CN114409725A. 2022.

[CR127] Yanqiu M, Yanling S, Wong, et al, inventor: Shenyang University of Chemical Technology, assignee. Oleanolic acid derivative with anti-tumor activity and preparation method thereof. CN114380884A. 2022.

[CR128] Yao N, Zeng C, Zhan T, He F, Liu M et al (2019) Oleanolic acid and ursolic acid induce UGT1A1 expression in HEPG2 cells by activating PXR rather than car. Front Pharmacol. 10.3389/fphar.2019.01111 31611795 10.3389/fphar.2019.01111 PMC6777376

[CR129] Yoo KH, Park JH, Cui EJ, Kim KI, Kim JY, Kim J, Chung IS (2012) 3-O-Acetyloleanolic acid induces apoptosis in human colon carcinoma Hct-116 cells. Phytother Res. 10.1002/ptr.4616 22359244 10.1002/ptr.4616

[CR130] Zhan Y, Wang K, Li Q, Zou Y, Chen B et al (2018) The novel autophagy inhibitor alpha-HEDERIN promoted paclitaxel cytotoxicity by increasing reactive oxygen species accumulation in non-small cell lung cancer cells. Int J Mol Sci. 10.3390/ijms19103221 30340379 10.3390/ijms19103221 PMC6214018

[CR131] Zhang P, Li H, Chen D, Ni J, Kang Y, Wang S (2007) Oleanolic acid induces apoptosis in human leukemia cells through caspase activation and poly (ADP-ribose) polymerase cleavage. Acta Biochim Biophys Sin. 10.1111/j.1745-7270.2007.00335.x 17928930 10.1111/j.1745-7270.2007.00335.x

[CR132] Zhang CX, Wang T, Ma JF, Liu Y, Zhou ZG et al (2017) Protective effect of CDDO-ethyl amide against high-glucose-induced oxidative injury via the Nrf2/HO-1 pathway. Spine J. 10.1016/j.spinee.2017.03.015 28343048 10.1016/j.spinee.2017.03.015

[CR133] Zhong Y, Li HN, Zhou L, Su HS, Cheng MS, Liu Y (2021) Synthesis and antitumor activity evaluation of oleanolic acid saponins bearing an acetylated L-arabinose moiety. Carbohydr Res. 10.1016/j.carres.2021.108311 33866267 10.1016/j.carres.2021.108311

[CR134] Zhou L, Wang Z, Yu S, Xiong Y, Fan J et al (2020a) CDDO-me elicits anti–breast cancer activity by targeting LRP6 and FZD7 receptor complex. J Pharmacol Exp Ther. 10.1124/jpet.119.263434 32015160 10.1124/jpet.119.263434

[CR135] Zhou W, Zeng X, Wu X (2020b) Effect of oleanolic acid on apoptosis and autophagy of SMMC-7721 hepatoma cells. Med Sci Monit. 10.12659/msm.921606 32424110 10.12659/MSM.921606 PMC7251962

[CR136] Zhu Y, Dong Y. inventors; Nanjing University of Technology, Peop. Rep. China. assignee. Synthesis of 3-O-β-D-glucopyranose(1->3)-βD-glucopyranose (1->3)- β-D-glucopyranose(1->3)oleanolic acid with antitumor activity. CN103483411A. 2014.

[CR137] Žiberna L, Šamec D, Mocan A, Nabavi BA, Farooqi A, Sureda A, Nabavi S (2017) Oleanolic acid alters multiple cell signaling pathways: implication in cancer prevention and therapy. Int J Mol Sci. 10.3390/ijms18030643 28300756 10.3390/ijms18030643 PMC5372655

[CR138] Zou W, Chen S, Liu X, Yue P, Sporn MB, Khuri FR, Sun SY (2007) c-FLIP downregulation contributes to apoptosis induction by the novel synthetic triterpenoid methyl-2-cyano-3, 12-dioxooleana-1, 9-dien-28-oate (CDDO-Me) in human lung cancer cells. Cancer Biol Ther. 10.4161/cbt.6.10.4763 18253090 10.4161/cbt.6.10.4763

[CR139] Zou Y, Yan C, Liu JC, Huang ZJ, Xu JY et al (2017) Synthesis and anti-hepatocellular carcinoma activity of novel O^2^ -vinyl diazeniumdiolate-based nitric oxide-releasing derivatives of oleanolic acid. Chin J Nat Med. 10.1016/s1875-5364(18)30009-8 29329650 10.1016/S1875-5364(18)30009-8

